# Vaccination induces broadly neutralizing antibody precursors to HIV gp41

**DOI:** 10.1038/s41590-024-01833-w

**Published:** 2024-05-30

**Authors:** Torben Schiffner, Ivy Phung, Rashmi Ray, Adriana Irimia, Ming Tian, Olivia Swanson, Jeong Hyun Lee, Chang-Chun D. Lee, Ester Marina-Zárate, So Yeon Cho, Jiachen Huang, Gabriel Ozorowski, Patrick D. Skog, Andreia M. Serra, Kimmo Rantalainen, Joel D. Allen, Sabyasachi Baboo, Oscar L. Rodriguez, Sunny Himansu, Jianfu Zhou, Jonathan Hurtado, Claudia T. Flynn, Katherine McKenney, Colin Havenar-Daughton, Swati Saha, Kaitlyn Shields, Steven Schultze, Melissa L. Smith, Chi-Hui Liang, Laura Toy, Simone Pecetta, Ying-Cing Lin, Jordan R. Willis, Fabian Sesterhenn, Daniel W. Kulp, Xiaozhen Hu, Christopher A. Cottrell, Xiaoya Zhou, Jennifer Ruiz, Xuesong Wang, Usha Nair, Kathrin H. Kirsch, Hwei-Ling Cheng, Jillian Davis, Oleksandr Kalyuzhniy, Alessia Liguori, Jolene K. Diedrich, Julia T. Ngo, Vanessa Lewis, Nicole Phelps, Ryan D. Tingle, Skye Spencer, Erik Georgeson, Yumiko Adachi, Michael Kubitz, Saman Eskandarzadeh, Marc A. Elsliger, Rama R. Amara, Elise Landais, Bryan Briney, Dennis R. Burton, Diane G. Carnathan, Guido Silvestri, Corey T. Watson, John R. Yates, James C. Paulson, Max Crispin, Gevorg Grigoryan, Andrew B. Ward, Devin Sok, Frederick W. Alt, Ian A. Wilson, Facundo D. Batista, Shane Crotty, William R. Schief

**Affiliations:** 1 Department of Immunology and Microbiology, The Scripps Research Institute, La Jolla, CA USA; 2 IAVI Neutralizing Antibody Center, The Scripps Research Institute, La Jolla, CA USA; 3 Center for HIV/AIDS Vaccine Immunology and Immunogen Discovery (CHAVD), The Scripps Research Institute, La Jolla, CA USA; 4 Institute for Drug Discovery, Leipzig University Medical Faculty, Leipzig, Germany; 5 Division of Vaccine Discovery, La Jolla Institute for Allergy and Immunology, La Jolla, CA USA; 6 The Ragon Institute of Mass General, MIT and Harvard, Cambridge, MA USA; 7 Department of Integrative Structural and Computational Biology, The Scripps Research Institute, La Jolla, CA USA; 8 Howard Hughes Medical Institute, Program in Cellular and Molecular Medicine, Boston Children’s Hospital, Boston, MA USA; 9 Department of Genetics, Harvard Medical School, Boston, MA USA; 10 School of Biological Sciences, University of Southampton, Southampton, UK; 11 Department of Molecular Medicine, The Scripps Research Institute, La Jolla, CA USA; 12 Department of Biochemistry and Molecular Genetics, University of Louisville School of Medicine, Louisville, KY USA; 13 Moderna, Inc., Cambridge, MA USA; 14 Department of Computer Science, Dartmouth College, Hanover, NH USA; 15 Division of Microbiology and Immunology, Emory National Primate Research Center, Emory University, Atlanta, GA USA; 16 Department of Microbiology and Immunology, Emory School of Medicine, Atlanta, GA USA; 17 Multi-omics Vaccine Evaluation Consortium, The Scripps Research Institute, La Jolla, CA USA; 18 San Diego Center for AIDS Research, The Scripps Research Institute, La Jolla, CA USA; 19 Department of Pathology and Laboratory Medicine, Emory University School of Medicine, Atlanta, GA USA; 20 Department of Biological Sciences, Dartmouth College, Hanover, NH USA; 21 Department of Biology, Massachusetts Institute of Technology, Cambridge, MA USA; 22 Division of Infectious Diseases, Department of Medicine, University of California San Diego, La Jolla, CA USA; 23 Present Address: Generate Biomedicines, Inc., Somerville, MA USA

**Keywords:** Vaccines, X-ray crystallography, HIV infections

## Abstract

A key barrier to the development of vaccines that induce broadly neutralizing antibodies (bnAbs) against human immunodeficiency virus (HIV) and other viruses of high antigenic diversity is the design of priming immunogens that induce rare bnAb-precursor B cells. The high neutralization breadth of the HIV bnAb 10E8 makes elicitation of 10E8-class bnAbs desirable; however, the recessed epitope within gp41 makes envelope trimers poor priming immunogens and requires that 10E8-class bnAbs possess a long heavy chain complementarity determining region 3 (HCDR3) with a specific binding motif. We developed germline-targeting epitope scaffolds with affinity for 10E8-class precursors and engineered nanoparticles for multivalent display. Scaffolds exhibited epitope structural mimicry and bound bnAb-precursor human naive B cells in ex vivo screens, protein nanoparticles induced bnAb-precursor responses in stringent mouse models and rhesus macaques, and mRNA-encoded nanoparticles triggered similar responses in mice. Thus, germline-targeting epitope scaffold nanoparticles can elicit rare bnAb-precursor B cells with predefined binding specificities and HCDR3 features.

## Abstract (web summary)

Schief and colleagues show that germline-targeting epitope scaffolds can elicit responses from rare broadly neutralizing antibody precursor B cells with predefined binding specificities and genetic features.

## Main

Broad vaccine protection against highly antigenically diverse viruses, such as human immunodeficiency virus (HIV), hepatitis C virus, influenza or the family of betacoronaviruses, has not been achieved in humans but will likely require induction of broadly neutralizing antibodies (bnAbs) that bind to conserved epitopes on otherwise variable membrane glycoproteins. Monoclonal bnAbs for each of the above pathogens have been discovered, and specific genetic and structural features of each bnAb allow binding to its cognate epitope^1–4^. To use known bnAbs as guides for the design of vaccines that elicit similar responses, strategies to induce bnAbs with predefined genetic properties and binding specificities are needed^5–7^. One such strategy, germline-targeting vaccine design, is predicated on molecular design of the ‘priming’ immunogen to first elicit responses from rare bnAb-precursor B cells with genetic properties needed for bnAb development. Following the prime, sequential boosting with immunogens of increasing similarity to the native glycoprotein aims to guide B cell maturation to produce bnAbs targeting the desired epitope^8–11^.

Germline-targeting priming in humans was demonstrated for the eOD-GT8 60mer immunogen targeting precursors for VRC01-class bnAbs specific for the HIV envelope CD4-binding site^11^, which was an advance toward the goal of developing precision vaccines that elicit prespecified classes of bnAbs. However, in contrast to the V_H_-dominant binding mode of VRC01-class bnAbs, most bnAbs to HIV and other viruses exhibit heavy chain complementarity determining region 3 (HCDR3)-dominant interactions with antigen, making it critical to demonstrate induction of HCDR3-dominant bnAb precursors by germline-targeting priming immunogens^7^. An effective HIV vaccine will need to induce several different classes of bnAbs for sufficient coverage against global isolates. Induction of HCDR3-dominant bnAbs to the membrane-proximal external region (MPER) of the HIV-1 envelope protein (Env) might be crucial due to the high breadth of neutralization provided by such bnAbs (for example, approximately 92–98% for bnAbs 10E8 (ref. ^12^), LN01 (ref. ^13^) and DH511 (ref. ^14^)), the relatively high epitope conservation that should reduce the potential of viral escape, and the strong protection by 10E8 in a passive nonhuman primate (NHP) immunization study despite relatively low potency against the challenge virus^15^. However, induction of MPER bnAbs faces challenges, including the recessed location of the MPER at the base of the Env trimer^12,16^, the need to induce antibodies with long HCDR3s bearing specific sequence motifs, and the lack of affinity of most MPER bnAb precursors for their peptide epitopes^17–19^. Furthermore, immune tolerance mechanisms block the induction of MPER bnAbs 2F5 and 4E10, potentially due to lipid reactivity^20^, raising concerns that other more potent MPER bnAbs, such as 10E8, might also face tolerance barriers^21–24^. Here, we developed and validated germline-targeting epitope scaffold nanoparticle priming immunogens to induce 10E8-class HCDR3-dominant bnAb-precursor responses. These immunogens represent candidates for human vaccination and demonstrate design and evaluation processes that could be applied to other bnAb targets.

## Results

### 10E8-class naive precursors are present in most humans

**Fig. 1:**
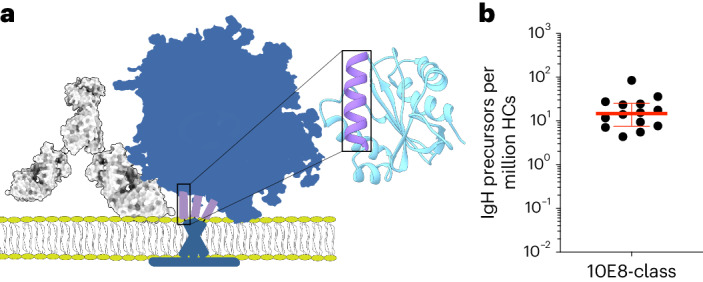
10E8-class bnAb precursors are present in most humans. **a**, Schematic of the epitope scaffold design showing antibody 10E8 (gray) and Env (blue), including the MPER (purple) that was grafted onto an unrelated epitope scaffold (cyan). **b**, Frequency of 10E8-class IgH precursors in 14 NGS datasets^7,27^ of heavy chains from HIV-seronegative humans defined as sequences with genes encoding V_H_ closely related to 10E8 and HCDR3 lengths of 21–24 aa with a YxFW motif at the correct position. Lines indicate the median and 25 and 75% quantiles; HCs, heavy chains.

**Extended Data Fig. 1:**
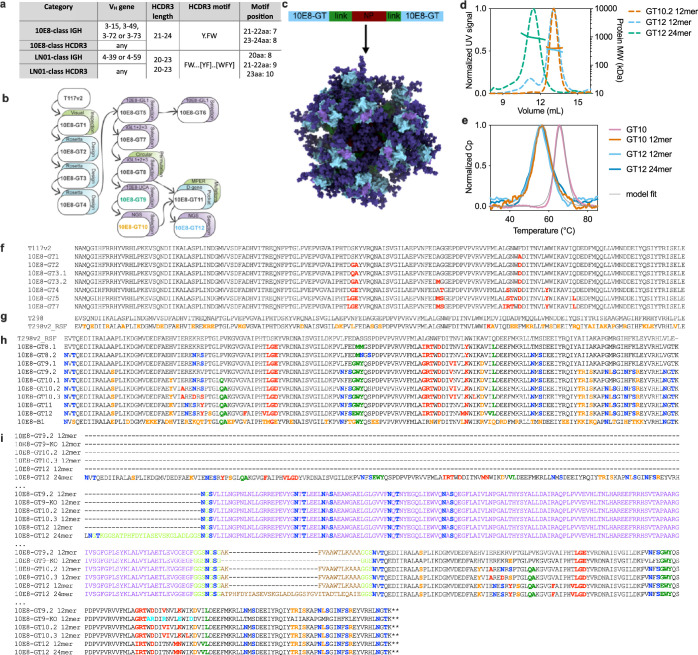
Design and properties of immunogens. **a**, Overview of 10E8-class and LN01-class antibody categories. HCDR3 motifs are shown as regular expressions that were used to query the database. If multiple amino acids were allowed at the same position, they are shown in square brackets; positions in which all amino acids were allowed are indicated as ‘.’. **b**, Schematic of the development of MPER-GT scaffolds. **c**, Schematic overview of nanoparticle formation by genetic fusion of the immunogen (T2983^−^GT) to each terminus of the 3-dehydroquinase nanoparticle from *Thermus thermophilus* (NP) via flexible linkers containing exogeneous T-help peptides derived from *Aquifex aeolicus* lumazine synthase (link). The epitope scaffold is shown in light blue, the MPER graft in purple, the linker in green, the nanoparticle in red and glycans in dark blue. **d**, SEC-MALS traces of 10E8-GT NPs. Normalized UV_280_ absorptions are shown as dotted lines and protein molecular weights of main peaks are shown as solid lines. **e**, DSC measurements of the indicated monomers and nanoparticles with results from a fit indicated in light grey. **f**, Amino acid sequences of 10E8-GT epitope scaffolds through generation 7, none of which had the circular permutation present in later generations. Germline-targeting mutations are highlighted in red; N-linked glycosylation sites are blue; the D-gene binding pocket is green; resurfacing and/or solubility enhancing mutations are orange. All sequences are succeeded by a 6x His-tag, unless the protein ends in stop codons (denoted by symbol *). 10E8-GT8.2 through 10E8-GT12 are preceded by a mammalian secretion signal. **g**, Resurfaced T298v2 sequences compared to previously published T298 32. Colors as in **f**. **h**, Amino acid sequences of monomeric immunogens based on resurfaced circularly permutated T298v2, with colors as in **f**. **i**, Amino acid sequences of multivalent nanoparticles. Sequences are wrapped over multiple lines. The 3-dehydroquinase is shown in purple; additional T help epitopes are brown; epitope KO mutations are cyan; and all other colors are shown as in **f**.

Structural^12,16,25,26^ and mutational^12,18^ data indicate that 10E8 binds to its MPER helical peptide epitope primarily through a germline D_H_3-3-encoded binding motif YxFW positioned near the tip of a long (22-amino acid (aa)) HCDR3, required to access the sterically occluded epitope at the base of full-length membrane-bound Env (Fig. 1a). The activity of 10E8 bnAb further requires a PP motif in the junction between D and J genes within the HCDR3, which could have arisen either during V(D)J recombination or somatic hypermutation (SHM), and germline-encoded HCDR1 and framework region 2 residues and somatically mutated HCDR2 residues within the gene encoding V_H_3-15. We therefore defined 10E8-class heavy chain precursors as heavy chains with a V_H_ gene closely related to V_H_3-15 and an HCDR3 length of 21–24 aa with a YxFW motif at the equivalent position within the HCDR3 as 10E8 (Extended Data Fig. 1a). This definition allowed for diverse V–D and D–J junctions and did not require the PP motif that can arise during SHM. To determine if heavy chains with these properties were present in humans, we searched an ultradeep next-generation sequencing (NGS) dataset of primarily naive IgM heavy chains from 14 HIV-seronegative donors^7,27^. Heavy chains matching the 10E8-class properties were found in all donors, with a geomean frequency of 1:68,000 (Fig. 1b).

The 10E8 light chain contributes to binding of membrane-associated Env by contacting the virion lipid membrane and conformationally stabilizing HCDR3 (ref. ^26^). The range of germline light chains that have the potential to acquire mutations to mediate such contacts is unclear but could be large. In two paired heavy chain–light chain datasets^28,29^, human light chains within the V_L_3 family used by 10E8 were paired with V_H_3-15 heavy chains at a frequency of approximately 1:7.5, suggesting that the frequency of 10E8-class heavy chain–light chain precursors was approximately 1:510,000. Thus, 10E8-class precursors are present in healthy humans at a substantial frequency.

### Germline-targeting immunogens bind 10E8-class precursors

Because the MPER region is sterically occluded at the base of full-length membrane-bound Env and absent from most soluble native-like trimers^30^, epitope scaffold immunogens were previously designed to conformationally stabilize and expose the C-terminal MPER helix^26,31,32^ (Fig. 1a). We prioritized one of these epitope scaffolds, T117v2 (ref. ^26^), for further optimization due to its favorable thermal stability, solubility and presentation of surfaces adjacent to the MPER graft that could be engineered to increase contacts with the YxFW motif in the 10E8 HCDR3. T117v2 bound strongly to mature 10E8 (*K*_d_ = 390 pM) but showed no binding (*K*_d_ ≥ 100 µM) to 52 10E8-class precursors identified in the NGS database search above paired with the inferred germline (iGL) 10E8 light chain (hereafter NGS precursors; Supplementary Table 1).

**Fig. 2:**
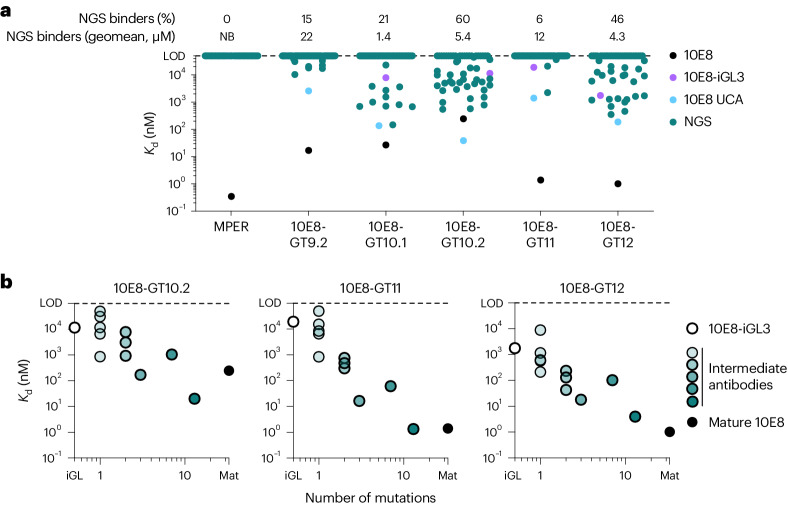
10E8-GT immunogens bind diverse 10E8-class precursors. **a**, SPR-measured monovalent *K*_d_ values for the scaffold without germline-targeting mutations (MPER) and various 10E8-GT scaffolds (10E8-GT9.2 to 10E8-GT12) binding to mature 10E8, germline-reverted 10E8 (10E8-iGL3), the proposed 10E8 UCA^18^ and multiple NGS-derived 10E8-class human precursor heavy chains paired with the germline-reverted 10E8 light chain (NGS). Each symbol represents a different antibody; LOD, limit of detection; NB, no binding. **b**, SPR-measured monovalent *K*_d_ values for 10E8-GT10.2, 10E8-GT11 and 10E8-GT12 binding to different antibodies containing the indicated number of 10E8-class mutations, including fully germline-reverted 10E8 (10E8-iGL3), partially mature 10E8-class antibodies (intermediates) and mature (Mat) 10E8.

We then performed a multistate design and selection process aimed at developing T117v2-based immunogens with the following features: 10 µM affinity or better for the 10E8 unmutated common ancestor (UCA) and as many NGS precursors as possible to enable priming of diverse 10E8-class precursors^7,11,33^; an affinity gradient for 10E8-class antibodies with the highest affinity for mature 10E8 to favor affinity maturation toward mature 10E8 in vivo^5,7,11,34^; multivalent display of epitope scaffolds on single-component self-assembling nanoparticles to facilitate mRNA lipid nanoparticle (mRNA-LNP) delivery, improve trafficking to lymph nodes^35^ and increase B cell responses^5^; and N-linked glycosylation sites added to the scaffold and base nanoparticle to reduce off-target responses^36^. Using a combination of structure-based design, computational modeling and directed evolution via yeast surface display^34^, we iteratively optimized binding of T117v2 to 10E8 iGL, UCA and NGS precursors, resulting in a series of immunogens that we refer to as 10E8-GT (Extended Data Fig. 1b–h and Methods). After nine rounds of optimization, 10E8-GT9.2 bound with low affinity to a small subset (15%) of NGS precursors (geomean *K*_d_ = 22 µM; Fig. 2a, Extended Data Fig. 1h, and Supplementary Tables 1 and 2). Further optimization of a pocket designed to contact germline D_H_3-3-encoded residues at the tip of the 10E8 HCDR3 that are critical for 10E8 neutralization^12^ produced 10E8-GT10.1 and 10E8-GT10.2. These bound to more NGS precursors (22% and 60%, respectively), with geomean *K*_d_ values of 1.4 µM and 5.4 µM, respectively (Fig. 2a), but compared with T117v2 they bound weakly to mature 10E8 (*K*_d_ = 27 nM and *K*_d_ = 247 nM, respectively; Fig. 2a). Optimization of affinity for mature 10E8 generated 10E8-GT11 that had high affinity (*K*_d_ = 1.4 nM) for mature 10E8 but low affinity (geomean *K*_d_ = 12 µM) for a minority (6%) of NGS precursors (Fig. 2a,b and Extended Data Fig. 1). Finally, we simultaneously optimized the binding of mature 10E8 and NGS precursors to produce 10E8-GT12 (Extended Data Fig. 1). This final design engaged 46% of precursors with a geomean *K*_d_ of 4.3 µM and bound strongly (*K*_d_ = 1.0 nM) to mature 10E8 (Fig. 2a,b).

**Extended Data Fig. 2:**
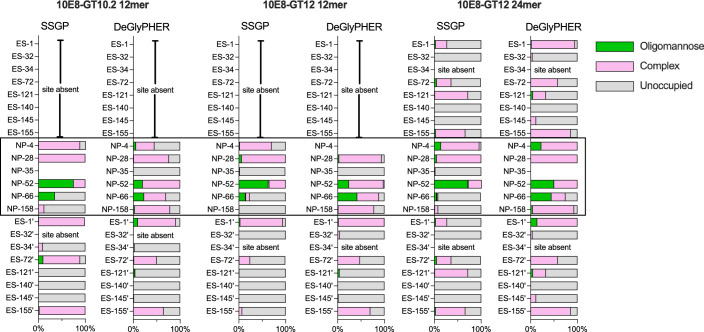
Glycosylation sites on 10E8-GT nanoparticles vary in occupancy. Site-specific glycan analysis was measured using the single site glycan profiling (SSGP) and DeGlyPHER methods. Positions of N-linked glycosylation sites are indicated as relative positions within the epitope-scaffold (ES) or the nanoparticle (NP, indicated with a black box). The 24mer contains two independent copies of the scaffold, which cannot be distinguished by either method and therefore averaged values are shown.

We multimerized 10E8-GT scaffolds by fusion to self-assembling nanoparticles from hyperthermophilic bacteria (Extended Data Fig. 1c–e,i). 10E8-GT10.2 12mer and 10E8-GT12 12mer, based on fusion to a glycan-shielded variant of the dodecameric 3-dehydroquinase from *Thermus thermophilus*, and 10E8-GT12 24mer, created by fusing 10E8-GT12 to each terminus of the 3-dehydroquinase nanoparticle protomer, were expressed with high yield (Extended Data Fig. 1c–e,i). We also added N-linked glycosylation sites to scaffold surfaces outside the MPER graft to reduce off-target responses^36^. Site-specific glycosylation analysis by mass spectrometry indicated that approximately 50% of glycosylation sites were occupied (Extended Data Fig. 2). We thus developed self-assembling nanoparticles presenting 10E8-GT scaffolds with broad affinity for 10E8-class precursors.

### Scaffold–antibody interactions mimic epitope and the HCDR3 motif

**Fig. 3:**
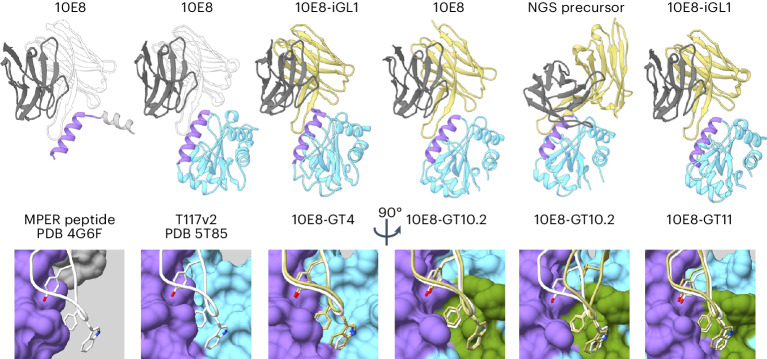
10E8-GT immunogens mimic the interaction between 10E8 and the MPER. Structures (from left to right) of 10E8 bnAb bound to MPER peptide^12^, 10E8 bnAb bound to T117v2 scaffold^26^, 10E8-iGL1 bound to 10E8-GT4 scaffold, 10E8 bnAb bound to 10E8-GT10.2 scaffold, NGS precursor 10E8-NGS-03 bound to 10E8-GT10.2 scaffold and 10E8-iGL1 bound to 10E8-GT11 scaffold, in which the previously published MPER peptide and T117v2 complexes with 10E8 are shown for comparison. Top, structures shown as cartoon diagrams, aligned on the MPER (purple), with antibody heavy chain in white or yellow, light chain in gray and scaffold in blue. Antibody constant regions are omitted for clarity. Bottom, interaction between the HCDR3 YxFW motif (sticks) and the engineered D_H_ binding pocket (green) on the scaffold. All structures were determined by crystallography, except for the complex of 10E8 bnAb with 10E8-GT10.2, which was determined by cryo-EM and included a scaffold-specific off-target Fab (not shown) to facilitate image reconstruction.

**Extended Data Fig. 3:**
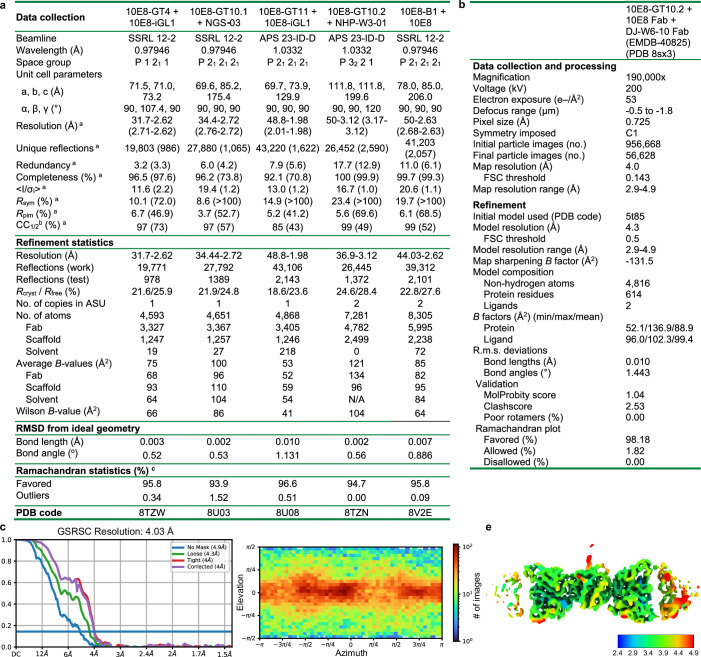
Data collection, refinement and validation. **a**, Data collection and refinement statistics of x-ray crystallography. ^a^ Numbers in parentheses refer to the highest resolution shell; ^b^ CC_1/2_ = Pearson correlation coefficient between two random half datasets; ^c^ From MolProbity^64^. **b**, Summary statistics of data collection, refinement and validation of the cryo-EM reconstruction of 10E8-GT10.2 in complex with 10E8 and W6-10 Fabs. **c**, Fourier Shell Correlation, **d**, angular sampling and **e**, map colored according to local resolution (units Ångstrom) of the cryo-EM reconstruction of 10E8-GT10.2 in complex with 10E8 and W6-10 Fabs.

To assess the structural mimicry of the MPER helix within the epitope scaffold and the interaction of the epitope scaffold with the 10E8-class D gene YxFW motif, we determined three crystal structures and one cryoelectron microscopy (cryo-EM) structure of 10E8-GT epitope scaffolds complexed with 10E8-class human antibodies (Fig. 3 and Extended Data Fig. 3). These included a 2.62-Å-resolution crystal structure of the early-stage design 10E8-GT4 bound to a variant of 10E8 iGL bearing the mature 10E8 HCDR3 (10E8-iGL1; complex 1), a 4.0-Å cryo-EM structure of 10E8-GT10.2 in complex with mature 10E8 and a scaffold-specific ‘off-target’ monoclonal antibody (mAb) to help image processing (complex 2), a 2.7-Å-resolution structure of nonglycosylated 10E8-GT10.1 in complex with the NGS precursor 10E8-NGS-03 (complex 3), and a 1.9-Å-resolution structure of 10E8-GT11 bound to 10E8-iGL1 (complex 4; Fig. 3 and Extended Data Fig. 3). In all four complexes, the overall structures of the epitope scaffold and the MPER helix were similar to the original T117v2 scaffold complexed with 10E8, with backbone root mean square deviation (bb-r.m.s.d.) values of 0.73, 0.89, 0.75 and 0.62 Å for the 10E8-GT antigens, respectively (Fig. 3). In all except complex 3, the antibody engaged the epitope scaffold at an angle closely resembling the interaction between mature 10E8 and the MPER peptide (Fig. 3), and the D gene YxFW motif interacted with the engineered D gene binding pocket and adopted a conformation similar to that of mature 10E8 bound to MPER peptide, with all-atom r.m.s.d. values computed over YxFW of 0.5, 0.47 and 0.32 Å for complexes 1, 2 and 4, respectively (Fig. 3). In complex 3, the FW portion of the YxFW motif also accurately mimicked the interaction between mature 10E8 and MPER peptide (all-atom r.m.s.d. = 1.04 Å over FW), but the Yx portion of the motif was divergent, owing to the antibody approaching the MPER from an angle differing from mature 10E8 by ~46°, potentially due to a different conformation of the immature HCDR3 (Fig. 3). In conclusion, despite variability in the angle of binding of diverse precursors, the 10E8 scaffolds stabilized the MPER in the 10E8-bound conformation and consistently engaged the hydrophobic tip of 10E8-class HCDR3s in a manner closely resembling the interaction of mature 10E8 with gp41.

### 10E8-GT scaffolds isolate 10E8-class human naive B cells

**Fig. 4:**
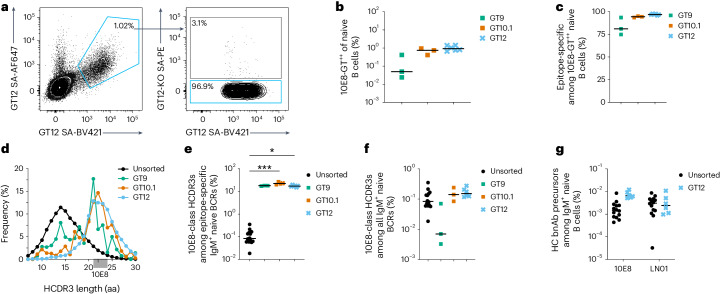
10E8-GT scaffolds engage 10E8-class HCDR3s in human blood. **a**, Representative flow cytometry staining of 10E8-GT12 double-positive (10E8-GT12^++^; signifying binding to two probes with different fluorochromes; left) and epitope-specific 10E8-GT12^++^10E8-GT12-KO^–^ (right) CD20^+^CD27^–^IgD^+^IgG^–^ naive B cells from HIV-seronegative donors; SA, streptavidin. **b**, Frequency of 10E8-GT9^++^ (*n* = 3 donors), 10E8-GT10.1^++^ (*n* = 3) and 10E8-GT12^++^ (*n* = 6) cells among CD20^+^IgG^–^ naive B cells for 10E8-GT9 and 10E8-GT10 or CD20^+^CD27^–^IgD^+^IgG^–^ naive B cells for 10E8-GT12 that were sorted from HIV-seronegative donors and BCR sequenced using either a Sanger sequencing method (squares) or a 10x Genomics sequencing method (crosses). **c**, Percentage of 10E8-GT9^++^, 10E8-GT10.1^++^ and 10E8-GT12^++^ naive B cells (CD20^+^IgG^−^ B cells or CD20^+^CD27^−^IgD^+^IgG^−^ B cells, as in **b**) that are epitope specific (10E8-GT9^++^10E8-GT9-KO^−^, 10E8-GT10.1^++^10E8-GT10.1-KO^−^ or 10E8-GT12^++^10E8-GT12-KO^−^). **d**, HCDR3 length distribution for human naive BCRs sorted by 10E8-GT9, 10E8-GT10.1 and 10E8-GT12 (average of all donors in each case). NGS datasets (*n* = 14) of heavy chains from HIV-seronegative humans served as unsorted controls^7,27^ where indicated (black). The targeted HCDR3 length range (21–24 aa) is highlighted in gray. The exact HCDR3 length for the 10E8 bnAb is indicated by a tick mark at 22 aa. **e**, Percentage of 10E8-class HCDR3s (with lengths of 21–24 aa and YxFW at the correct position within HCDR3) among epitope-specific (10E8-GT9^++^10E8-GT9-KO^−^, *n* = 3 donors; 10E8-GT10.1^++^10E8-GT10.1-KO^−^, *n* = 4 donors; 10E8-GT12^++^10E8-GT12-KO^−^ *n* = 6 donors) IgM^+^ BCRs compared to unsorted controls defined in **d**; *P = 0.03 and ***P = 0.0004. Data were analyzed by Kruskal–Wallis test with a Dunn’s multiple comparison correction. **f**, Percentage of 10E8-class HCDR3s among all naive IgM^+^ B cells compared to unsorted controls as in **e**. **g**, Percentage of 10E8-class and LN01-class IgH precursors among naive IgM^+^ B cells sorted with 10E8-GT12 or unsorted controls defined in **d**. Lines indicate median values.

**Extended Data Fig. 4:**
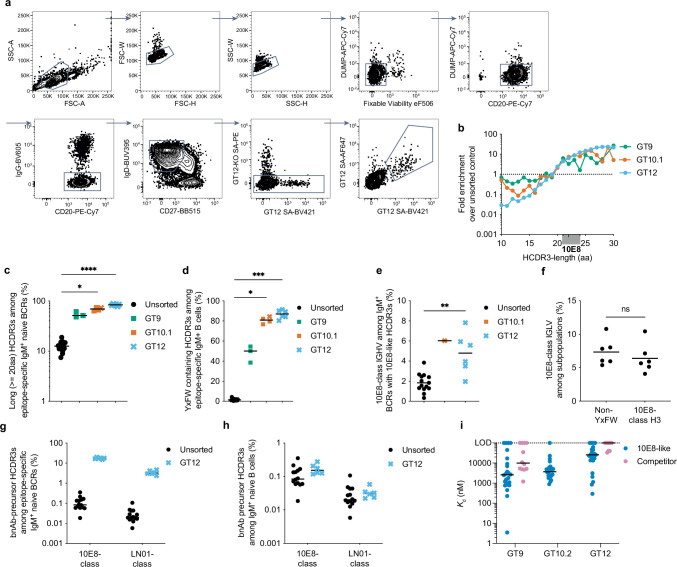
Ex vivo evaluation of 10E8-GT scaffolds, related to Fig. 4. **a**, Representative gating scheme. **b**, Enrichment of HCDR3 lengths among epitope-specific B cells over unsorted controls as in Fig. 4d. **c**, Frequency of long (>=20aa) HCDR3s among epitope-specific B cells (sorted) as in **b**, or among total IgM+ naïve B cells (unsorted). Symbols represent n = 3 (GT9), n = 4 (GT10.1), n = 6 (GT12) or n = 14 (unsorted controls) independent donors. * p = 0.046, **** p < 0.0001, Kruskal-Wallis test with Dunn’s multiple comparison correction. **d**, Percentage of HCDR3s containing the YxFW motif among epitope-specific B cells as in **c**. * p = 0.03, *** p = 0.0001, Kruskal-Wallis test with Dunn’s multiple comparison correction. **e**, Percentage of 10E8-class V_H_ among epitope-specific BCRs with 10E8-like HCDR3s (with length 21-24 aa and YxFW at correct position within HCDR3) within datasets obtained using the 10x Genomics sequencing method as in **c**. **p = 0.002, two-sided Mann-Whitney test. **f**, Percentage of IGVL3 family light chains among 10E8-GT12-sorted BCRs that are either 10E8-class (10E8-class H3) or lack the YxFW motif (non-YxFW). n = 6 independent donors, ns not significant, two-sided Wilcoxon test. **g**, Percentage of 10E8-GT12-specific naive IgM^+^ BCRs with 10E8-class or LN01-class HCDR3s. Symbols represent n = 6 (GT12) or n = 14 (unsorted controls) independent donors. **h**, Frequency of 10E8-class or LN01-class B cells among IgM+ naive B cells, detected through 10E8-GT12 sorting as in **g**. **i**, SPR-measured monovalent *K*_d_ values for 10E8-GT9, 10E8-GT10.2, and 10E8-GT12 monomer binding to 10E8-class and non-10E8-class (competitor) antibodies isolated by the respective scaffolds. Symbols represent different antibodies; lines represent median values.

To assess the repertoire of bona fide human naive B cell receptors (BCRs) able to respond to the 10E8-GT immunogens^7,34,37^, we characterized naive BCRs from the blood of HIV-seronegative human donors that bound the 10E8-GT epitope scaffolds (Extended Data Fig. 4a). On average, 10E8-GT9.2, 10E8-GT10.1 and 10E8-GT12 bound to 0.05%, 0.8% and 0.7% of naive CD20^+^CD27^–^IgD^+^IgG^–^ B cells (hereafter naive B cells), respectively (Fig. 4a,b). Among the epitope scaffold-binding naive B cells, 81%, 94% and 97% did not bind matching 10E8 epitope-knockout (KO) versions of the respective 10E8-GT constructs and are hereafter referred to as ‘epitope-specific BCRs’ (Fig. 4c and Extended Data Fig. 4a). BCR sequencing indicated that, compared to unsorted control datasets^28,29^, epitope-specific BCRs were highly enriched for long (≥20 aa) HCDR3s, comparable to the 22-aa-long HCDR3 of 10E8, with enrichments of 5.5-fold and 6.7-fold for 10E8-GT10.1 and 10E8-GT12, respectively (Fig. 4d, Extended Data Fig. 4b,c and Supplementary Table 3). Furthermore, epitope-specific BCRs were enriched for the crucial 10E8 D gene-encoded binding motif YxFW in the HCDR3, with 47%, 81% and 87% of epitope-specific BCRs containing the motif for 10E8-GT9.2, 10E8-GT10.1 and 10E8-GT12, respectively, compared to 1.4% for unsorted BCRs (Extended Data Fig. 4d). Overall, 18%, 23% and 16% of epitope-specific BCRs sorted with 10E8-GT9.2, 10E8-GT10.1 and 10E8-GT12, respectively, fulfilled all criteria of 10E8-class HCDR3s (Fig. 4e). Among all human naive B cells, those that bound 10E8-GT10.1 or 10E8-GT12 and had 10E8-class HCDR3s were surprisingly frequent (0.14% and 0.15%, respectively; Fig. 4f). Among the BCRs with 10E8-class HCDR3s, genes encoding 10E8-like V_H_were found with frequencies of 6.0% and 4.8% for 10E8-GT10.2 and 10E8-GT12, respectively, indicating an enrichment of 10E8-like IgH over the 1.7% frequency of such sequences found in unsorted control datasets (Extended Data Fig. 4e). 10E8-class light chain V, which is not expected to directly interact with 10E8-GT12, was found at a similar frequency among 10E8-class and non-10E8-class antibodies (Extended Data Fig. 4f).

We also searched 10E8-GT-binding sequences for signatures of other MPER bnAb lineages, including precursors of LN01-class MPER bnAbs, which are genetically and structurally distinct from 10E8-class bnAbs but share key features such as an D_H_3-3-encoded ‘FW’ motif at the tip of a long (20-aa) HCDR3 (ref. ^13^). Although immunogens were optimized for engagement of 10E8-class precursors, we detected LN01-class HCDR3s in all six samples of 10E8-GT12-sorted naive IgM^+^ B cells, with a median frequency of 3.3% (Extended Data Fig. 4g,h). Accounting for HCDR3 properties, the V_H_4-39 gene of LN01 and the frequency of epitope-specific B cells, the total frequency of 10E8-GT12-binding LN01-class IgH precursors among naive B cells was 1:41,000, which was only slightly lower than the frequency of 10E8-like IgH precursors (1:15,000; Fig. 4g).

**Extended Data Fig. 5:**
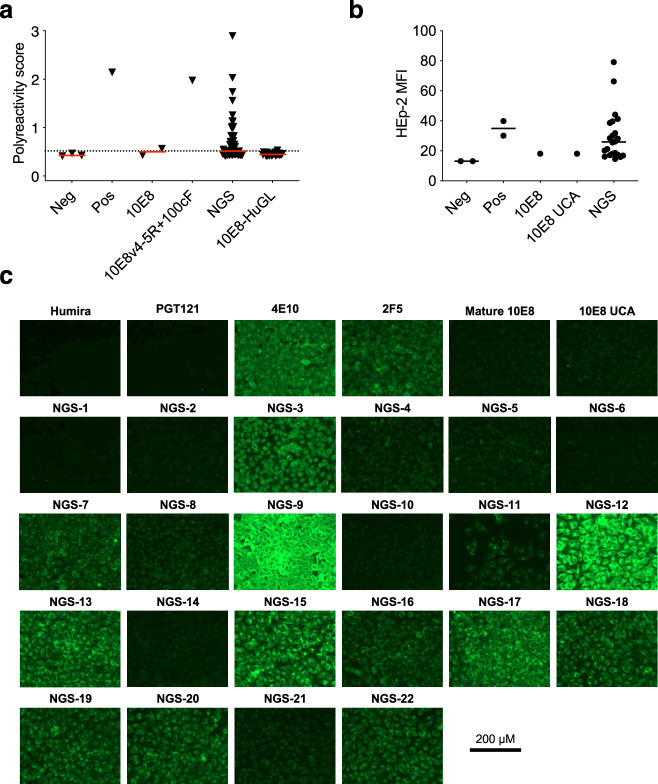
Poly- and Auto-reactivity of 10E8-class precursors. **a**, Polyspecificity reagent binding as measured by ELISA. PGT121, VRC01 and PGT128 served as negative controls, MPER bnAb 4E10 as a positive control. NGS-1 through −22 correspond to human NGS precursors described in the main text. These are 10E8-class heavy chain (HC) precursors identified from searching next-generation sequencing (NGS) datasets of primarily naive IgM HCs from 14 HIV-seronegative human donors^5,27^ paired with the inferred-germline 10E8 light chain (LC); 10E8-HuGL: bona fide HC/LC pairs isolated by epitope-specific sorting of naïve human B cells (n = 25). **b**, Mean fluorescence intensity (MFI) of antibodies as in **a** in a HEp-2 cell autoreactivity assay. **c**, raw images of data shown in **b**.

To evaluate the properties of the 10E8-GT-binding BCRs, we synthesized and expressed mAbs from B cells sorted with 10E8-GT9, 10E8-GT10.1 or 10E8-GT12 that had either 10E8-class or non-10E8-class HCDR3s. Surface plasmon resonance (SPR) analysis showed that 60 of 70 10E8-like mAbs had detectable affinity (*K*_d_ < 100 µM) for the respective sorting probe, and a minority (10 of 70) bound with submicromolar affinity (Extended Data Fig. 4i). Encouragingly, 10E8-class mAbs bound better to their respective sorting probe than non-10E8-class mAbs (Extended Data Fig. 4i). None of 25 bona fide human naive 10E8-class BCRs that were tested for in vitro polyreactivity registered positive, whereas 49% of 10E8-class NGS precursors with artificial heavy chain–light chain pairing were polyreactive (Extended Data Fig. 5). In summary, 10E8-GT scaffolds selectively engage human naive BCRs with 10E8-class HCDR3s and bind them with affinities shown to allow efficient B cell activation in vivo^7,11,33,38,39^.

### 10E8-class B cells function in vivo

**Fig. 5:**
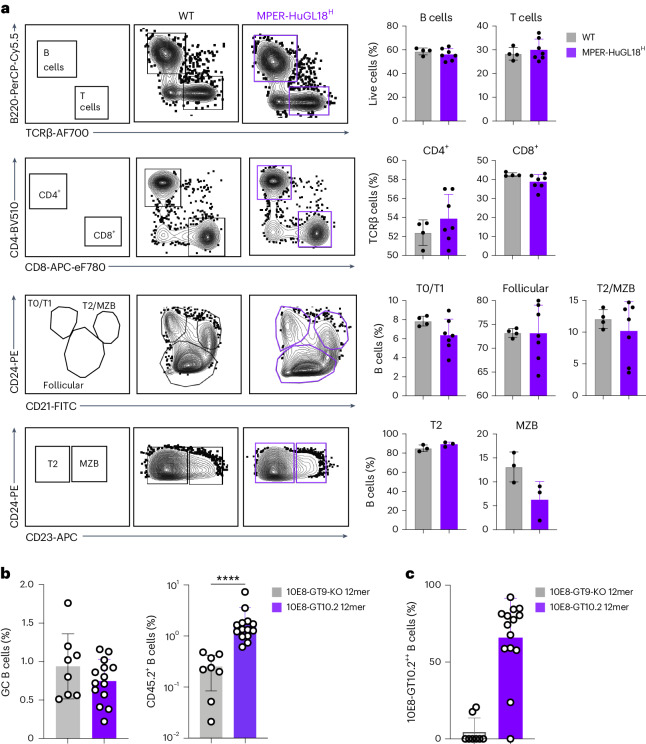
10E8-class B cells function in vivo. **a**, Flow cytometry analysis and quantification of B220^+^TCRβ^–^ B cells, B220^–^TCRβ^+^ total T cells, CD4^+^CD8^−^ T cells, CD4^−^CD8^+^ T cells, CD2^−^CD24^hi^ T0/T1 B cells, CD21^lo^CD24^lo^ follicular B cells, CD21^hi^CD24^hi^CD23^−^ T2 B cells and CD21^hi^CD24^hi^CD23^+^ marginal zone B (MZB) cells in the spleens of MPER-HuGL18^H^ mice (*n* = 7) compared to wild-type (WT) C57BL/6 mice (*n* = 4). Symbols represent individual animals, and error bars indicate standard deviation. **b**, Frequency of CD38^lo^CD95^+^ GC B cells among total B220^+^ B cells (left) and CD45.2^+^ B cells among CD38^lo^CD95^+^ GC B cells (right) at day 21 after immunization with 10E8-GT10.2 12mer (*n* = 14) or control 10E8-GT9-KO 12mer (*n* = 8) in CD45.1 wild-type recipient mice adoptively transferred with 200,000 CD45.2 MPER-HuGL18^H^ B cells. Symbols represent individual animals; bars indicate mean ± s.d.; ****P < 0.0001. Data were analyzed by two-sided Mann–Whitney test**. c**, Frequencies of 10E8-GT10^++^ cells among CD38^lo^CD95^+^CD45.1^−^CD45.2^+^ MPER-HuGL18^H^ GC cells as in **b**.

**Extended Data Fig. 6:**
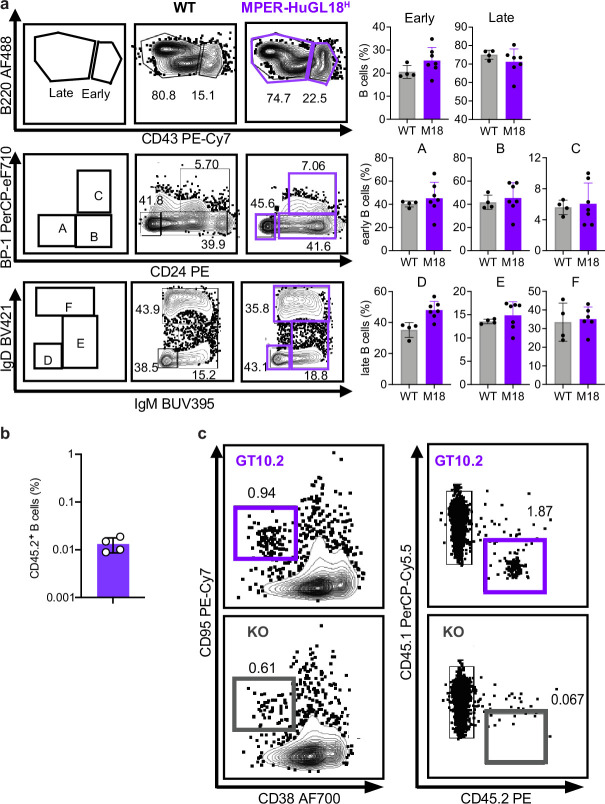
Immunization of MPER-HuGL18^H^ B cell adoptive transfer recipient mice with 10E8-GT10.2 12mers. **a**, Flow cytometry analysis of bone marrow cells from WT (n = 4) and MPER-HuGL18^H^ (M18, n = 7) mice; gating strategy shown on the left. B-cell progenitors (B220^+^) were divided into immature (CD43^+^) and mature (CD43^−^) cells. Early (CD43^+^) B-cell progenitors were subdivided into Hardy populations A (CD24^−^BP-1^−^), B (CD24^+^BP-1^−^), and C (CD24^+^BP1^+^). Late (CD43^−^) B-cell progenitors were subdivided into Hardy populations D (IgM^−^IgD^−^), E (IgM^+^IgD^int^), and F (IgM^+^IgD+). Right bars represent quantifications of these populations, error bars indicate SD. **b**, Frequency of CD45.2^+^ B cells among splenic B cells, one day after adoptive transfer of 200,000 CD45.2^+^ MPER-HuGL18^H^ B cells into CD45.1^+^ WT mice. Symbols represent individual animals, error bars indicate SD. **c**, Germinal center (GC) response to immunization in CD45.1^+^ WT mice adoptively transferred with 2 × 10^5^ CD45.2^+^ MPER-HuGL18^H^ B cells on Day 21 after immunization with 10E8-GT10.2 12mer or negative control 10E8-GT9-KO 12mer (KO). Left column shows the frequency of total GC (CD38^lo^CD95^+^) among B cells gated from SSL; right column shows the frequency of CD45.2 B cells among total GC.

To evaluate 10E8-class B cell development and activation in vivo, we created *IgH* knock-in (KI) mice using one of the highest-affinity human 10E8-class naive precursors, MPER HuGL18, identified through sorting human naive B cells that bound to 10E8-GT9.2 (Supplementary Table 1). B cell development in the bone marrow of *IgH*^MPER-HuGL-18/WT^*IgK*^WT/WT^ mice (hereafter MPER-HuGL18^H^ mice) was similar to that in wild-type mice (Extended Data Fig. 6a), and frequencies of Live/Dead^−^TCRβ^−^B220^+^ B cells, other B cell subpopulations and T cells among splenic lymphocytes in heterozygous MPER-HuGL18^H^ mice were comparable to those in wild-type mice (Fig. 5a). These data indicated normal B cell development in MPER-HuGL18^H^ mice, in contrast to previously developed MPER bnAb *IgH* or *IgHK* KI mice^22–24^.

To test the immunogenicity of 10E8-GT immunogens in mice with low frequencies of 10E8-class precursor B cells, we adoptively transferred CD3ε^–^CD4^–^CD8a^–^CD49b^–^Gr-1^–^Ter119^–^ B cells (hereafter B cells, unless otherwise specified) from CD45.2 MPER-HuGL18^H^ mice into CD45.1 wild-type recipient mice that lacked a suitable D_H_ gene encoding the YxFW motif^40^ and therefore could not generate 10E8-class responses. One day after transfer, the frequency of MPER-HuGL18^H^ CD45.2^+^ B cells was 0.013% of all splenic B cells (Extended Data Fig. 6b), approximately 11-fold lower than the frequency of B cells with 10E8-class HCDR3s in humans. One day after transfer, recipient mice were immunized with 10E8-GT10.2 12mer or the control immunogen 10E8-GT9-KO 12mer in alum adjuvant. At day 21 after immunization, both immunogens induced similar overall CD38^lo^CD95^+^ germinal center (GC) responses in the spleen (Fig. 5b and Extended Data Fig. 6c), but 10E8-GT10.2 12mer induced significantly higher frequencies of CD45.2^+^ MPER-HuGL18^H^ cells among GC B cells (Fig. 5b,c). Thus, MPER-HuGL18^H^ B cells developed normally and participated in GC responses after immunization with 10E8-GT10.2 12mer.

### mRNA-LNP vaccination induces diverse 10E8-class B cells

**Fig. 6:**
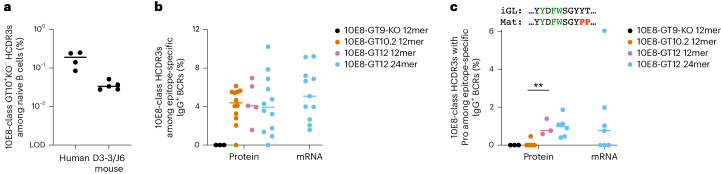
mRNA-LNP delivery of 10E8-GT12 nanoparticles primes diverse 10E8-class B cells. **a**, Percentage of 10E8-GT10.1^++^10E8-GT10.1-KO^–^ (epitope-specific) CD19^+^IgD^+^ naive B cells with 10E8-class HCDR3s for humans (as in Fig. 4f) and hD3-3/J_H_6 mice. **b**, Percentage of 10E8-GT9^–^KO^++^10E8-GT9^–^, 10E8-GT10.1^++^10E8-GT10.1-KO^–^ or 10E8-GT12^++^10E8-GT12-KO^–^ epitope-specific IgG^+^ BCRs with 10E8-class HCDR3s from day 42 after immunization of hD3-3/J_H_6 mice with 10E8-GT9-KO 12mer (*n* = 3), 10E8-GT10.2 12mer (*n* = 12), 10E8-GT12 12mer (*n* = 5) or 10E8-GT12 24mer (*n* = 12) delivered as protein in SMNP, respectively, or 10E8-GT12 24mer delivered by mRNA (*n* = 11). Symbols represent individual animals, and bars indicate median values. **c**, Percentage of epitope-specific IgG^+^ BCRs as in **b** with 10E8-class HCDR3s and at least one proline in position +7 or +8 relative to the YxFW motif from hD3-3/J_H_6 mice with >100 sequences. Sequences of genes encoding D_H_ in mature 10E8 and iGL are shown with the YxFW motif in green, and the targeted prolines are colored red; **P = 0.006. Data were analyzed by two-sided Mann–Whitney test.

**Extended Data Fig. 7:**
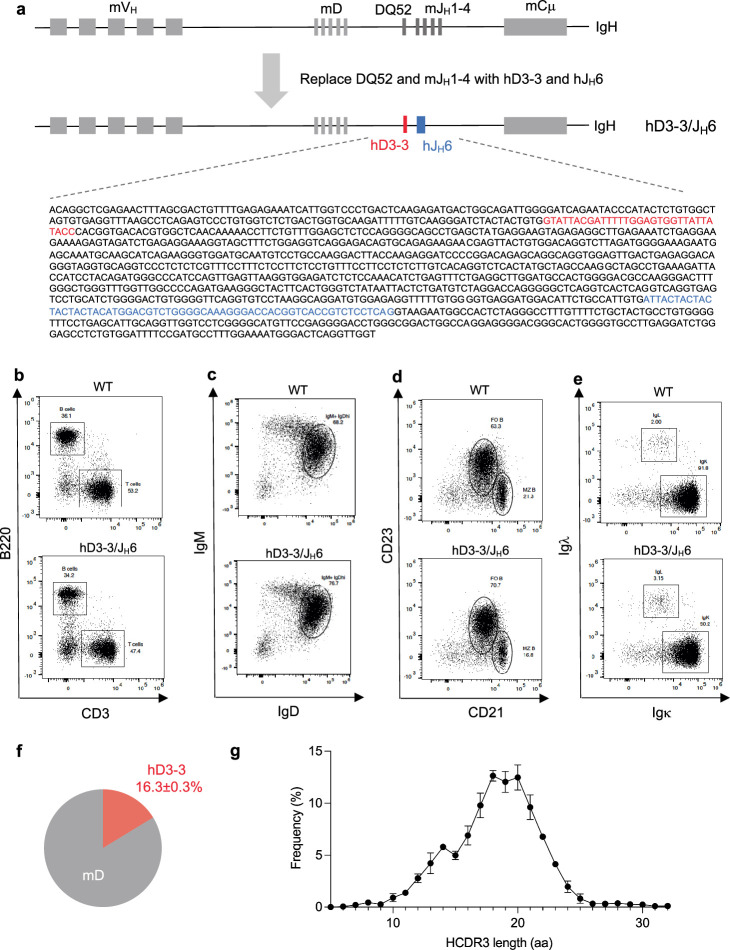
Generation and characterization of hD3-3/J_H_6 mice. **a**, Illustration of genetic modifications in hD3-3/J_H_6 mice (not drawn to scale) with hD3-3 and hJ_H_6 segments replacing mouse DQ52 and J_H_1-4 segments. Sequences of hD3-3 (in red), J_H_6 (in blue) and flanking regions are shown below the diagram. **b**, Characterization of B220^+^ B cell and CD3^+^ T cell populations among lymphocyte/live cell/single cells from homozygous D3-3/J6 mouse spleens by flow cytometry compared to a wild-type 129SVE mouse (WT). **c**, Characterization of IgM^+^IgD^hi^ naïve B cells among B cells as in **b**. **d**, Characterization of CD23^hi^CD21^low^ follicular (FO) B cells and CD23^low^CD21^hi^ marginal zone (MZ) B cells among lymphocyte/live cell/single cell/B220^+^CD93^low^ mature B cells, as in **b**. **e**, Characterization of Igk^+^ and Igl^+^ B cells among B cells as in **b**. **f**, Fraction of productive IgH rearrangements that contain hD3-3 or mouse D (mD), based on repertoire analysis of three homozygous hD3-3/J_H_6 mice. The hD3-3 usage value represents average ± standard deviation. Since hJ_H_6 is the sole J_H_ segment in homozygous hD3-3/J_H_6 mice, all V(D)J recombination events contain hJ_H_6. Therefore, 16.3% productive rearrangements in homozygous hD3-3/J_H_6 mice contain both hD3-3 and hJ_H_6. **g**, HCDR3 length distribution of productive IgH rearrangements that contain hD3-3 and J_H_6 as in **f**. Error bars indicate SD of three independent mice.

**Extended Data Fig. 8:**
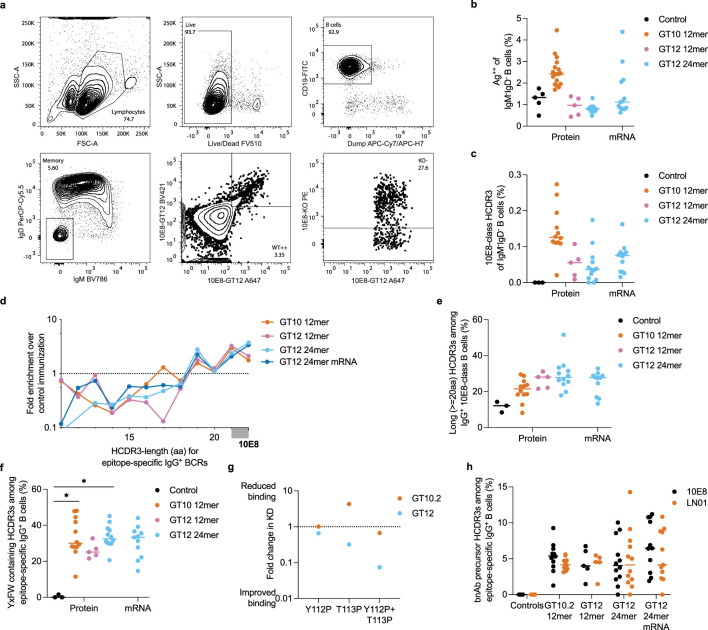
Immunogenicity of 10E8-GT nanoparticles in hD3-3/J_H_6 mice. **a**, Representative gating strategy of splenic B cells sorted for sequencing of BCRs from hD3-3/J_H_6 mice six weeks after immunization with 10E8-GT12 24mer. **b**, Percentage of 10E8-GT-binding cells (10E8-GT9-KO^++^, 10E8-GT10.1^++^ or 10E8-GT12^++−^) among IgM^−^IgD^−^ B cells, 42 days after immunization of hD3-3/J_H_6 mice with 10E8-GT9-KO 12mer (control, n = 3), 10E8-GT12 12mer (n = 12), 10E8-GT12 12mer (n = 5) or 10E8-GT12 24mer (n = 12) delivered as protein in SMNP, respectively, or 10E8-GT12 24mer delivered by mRNA (n = 11). Each symbol indicates an animal, lines indicate median values. **c**, Percentage of 10E8-class HCDR3s among all IgM^−^IgD^−^ B cells after immunization as in **b**. **d**, Enrichment ratio for HCDR3 amino acid (aa) length distribution for epitope-specific (10E8-GT10.1^++^10E8-GT10.1-KO^−^ or 10E8-GT12^++^10E8-GT12-KO^−^) IgG^+^ B cells from animals immunized with the indicated 10E8-GT immunogens, relative to HCDR3 amino acid length distribution for epitope-specific (10E8-GT9^−^KO^++^10E8-GT9^−^) IgG^+^ B cells from animals immunized with 10E8-GT9-KO 12mer as in **b**. HCDR3 lengths longer than 22 were only found in the 10E8-GT-immunized groups, precluding calculation of enrichment scores for longer HCDR3s. **e**, Frequency of long (>=20 aa) HCDR3s among epitope-specific IgG^+^ B cells as in **d**. **f**, Percentage of HCDR3s containing the YxFW motif among epitope-specific IgG^+^ B cells as in **c**. *p < 0.05, Kruskal-Wallis test with Dunn’s multiple comparison correction. P value (control vs. GT10 12mer): 0.02, p value (control vs. GT12 24mer): 0.01. **g**, Fold-change in SPR-measured K_D_ for 10E8-GT immunogens binding to 10E8-iGL3 upon addition of the indicated HCDR3 mutations. **h**, Percentage of epitope-specific (10E8-GT9-KO^++^10E8-GT9^−^,10E8-GT10.1^++^10E8-GT10.1-KO^−^, or 10E8-GT12^++^10E8-GT12-KO^−^) with 10E8^−^class or LN01-class HCDR3s among IgG^+^ BCRs from day 42 after immunization of hD3^−^3/J_H_6 as in **b**. Symbols represent individual animals; bars indicate median values.

To determine if 10E8-GT immunogens induced 10E8-class responses in a mouse with diverse 10E8-class precursors, we developed hD3-3/J_H_6 mice, in which human D_H_3-3 and J_H_6 segments replaced mouse DQ52 and JH1–JH4 segments, respectively (Extended Data Figs. 7 and 8a). BCR sequencing of homozygous hD3-3/J_H_6 naive CD19^+^IgD^+^ B cells specific for the MPER epitope on 10E8-GT10.2 found diverse 10E8-class HCDR3s at a frequency of 1:3,000 (Fig. 6a), 5.7-fold lower than in humans (1:525; Fig. 4f), indicating that induction of 10E8-class responses was more challenging in hD3-3/J_H_6 mice than in humans. Six weeks after a single immunization of hD3-3/J_H_6 mice with adjuvanted proteins 10E8-GT10.2 12mer, 10E8-GT12 12mer, 10E8-GT12 24mer, control 10E8-GT9-KO 12mer and mRNA-LNP-encoded 10E8-GT12 24mer, we sequenced immunogen-specific BCRs of IgM^–^IgD^–^ B cells (Extended Data Fig. 8a,b and Supplementary Table 4). Except for 10E8-GT9-KO 12mer, all immunogens induced 10E8-class HCDR3s (Fig. 6b and Extended Data Fig. 8c) and enriched for long HCDR3s (Extended Data Fig. 8d,e) and the YxFW motif (Extended Data Fig. 8f), whereas the frequencies of 10E8-class IgG^+^ BCRs were similar in all 10E8-GT-immunized groups (Fig. 6b). We also measured the frequency of prolines at positions +7 and +8 from the Y in the YxFW motif, which are important for neutralizing activity^12^. The addition of proline to either position in 10E8-iGL3 improved binding to 10E8-GT12 in vitro, whereas binding to 10E8-GT10.2 was unchanged or reduced (Extended Data Fig. 8g). In all mice immunized with 10E8-GT12 12mer for which sufficient sequencing data (>100 sequences) were available, we detected 10E8-like HCDR3s that contained at least one proline at position +7 or +8 (Fig. 6c), indicating that 10E8-GT12 nanoparticles selected for features required for neutralizing activity. These proline mutations were detected less frequently in mice immunized with 10E8-GT10 12mer (Fig. 6c).

All hD3-3/J_H_6 mice immunized with 10E8-GT10.2 12mer and 10E8-GT12 12mer and 11 of 12 hD3-3/J_H_6 mice immunized with 10E8-GT12 24mer produced detectable LN01-class HCDR3s, with median frequencies of 4.2%, 4.5% and 4.1% among epitope-specific BCRs, respectively (Extended Data Fig. 8h). Thus, 10E8-GT12 nanoparticles delivered as protein or mRNA-LNPs elicit responses from rare and diverse 10E8- and LN01-class precursors in vivo and select for additional 10E8-class features in the HCDR3.

### 10E8-GT 12mer induces 10E8-class BCRs in rhesus macaques

**Fig. 7:**
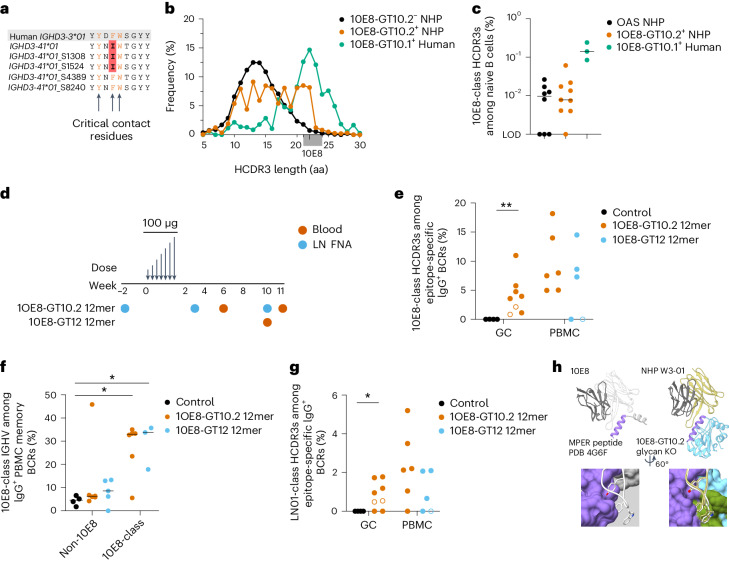
10E8-GT immunogens induce 10E8-class responses in NHPs. **a**, Alignment of known rhesus macaque homologs of the human gene encoding D_H_3-3. Macaque D-gene residues that differ from the critical YxFW binding motif (orange) that directly contacts the 10E8 epitope are highlighted in red. **b**, HCDR3 length distribution for 10E8-GT10.2^++^10E8^–^GT10.2-KO^–^ epitope-specific CD20^+^IgG^–^ naive B cells sorted from unimmunized macaques (*n* = 9) compared to 10E8-GT10.2^**−**^ nonbinding BCRs from the same macaques and the human naive BCRs, as in Fig. 4. **c**, Percentage of 10E8-class HCDR3s among CD20^+^IgG^−^ naive B cells from unimmunized macaques (*n* = 9) compared to human naive BCRs from Fig. 4 (*n* = 3) and to rhesus macaque sequences from the Observed Antibody Space (OAS) repository (*n* = 8)^49^. **d**, Macaque immunization schedule for an escalating dose of 10E8-GT10.2 12mer (*n* = 8) or 10E8-GT12 12mer (*n* = 6), indicating analysis of lymph node fine needle aspirates (LN FNA) at weeks –2, 3 and 10 and analysis of blood at weeks 6 and 11 or week 10 for macaques immunized with 10E8-GT10.2 12mer or 10E8-GT12 12mer, respectively. **e**, Percentage of 10E8-class HCDR3s among 10E8-GT10.2^++^10E8-GT10.2-KO^−^ or 10E8-GT12^++^10E8-GT12-KO^−^ epitope-specific IgG^+^ BCRs from macaques after immunization as in **d** in the GCs and PBMCs at all time points indicated in **d** combined for each macaque compared to macaques immunized with stabilized soluble HIV Env^50^ (control) at weeks 3, 4, 7 and 10 after immunization. Open symbols represent macaques lacking a permissive D_H_3-41 allele; **P = 0.004. Data were analyzed by two-sided Mann–Whitney test. **f**, Percentage of 10E8-class V_H_ among IgG^+^CD20^+^IgD^−^ memory BCRs with 10E8-class HCDR3s or among IgG^+^ CD20^+^IgD^−^ memory BCRs lacking the YxFW motif (non-10E8) from macaques after immunization as in **d**; *P < 0.05. Data were analyzed by Kruskal–Wallis test with a Dunn’s multiple comparison correction; control versus 10E8-class induced by 10E8-GT10.2 12mer, *P* = 0.03; control versus 10E8-class induced by 10E8-GT12 12mer, *P* = 0.02. **g**, Percentage of LN01-class HCDR3s among 10E8-GT10.2^++^10E8-GT10.2-KO^−^ or 10E8-GT12^++^10E8-GT12-KO^−^ epitope-specific IgG^+^ BCRs from macaques after immunization as in **d**; *P = 0.04. Data were analyzed by two-sided Mann–Whitney test. **h**, Crystal structure of a 10E8-GT10.2-induced macaque antibody with a YxIW motif from week 3 in complex with 10E8-GT10.2 (right) with the structure of 10E8 bound to peptide^12^ (left) shown for reference. Colors are as in Fig. 3.

**Extended Data Fig. 9:**
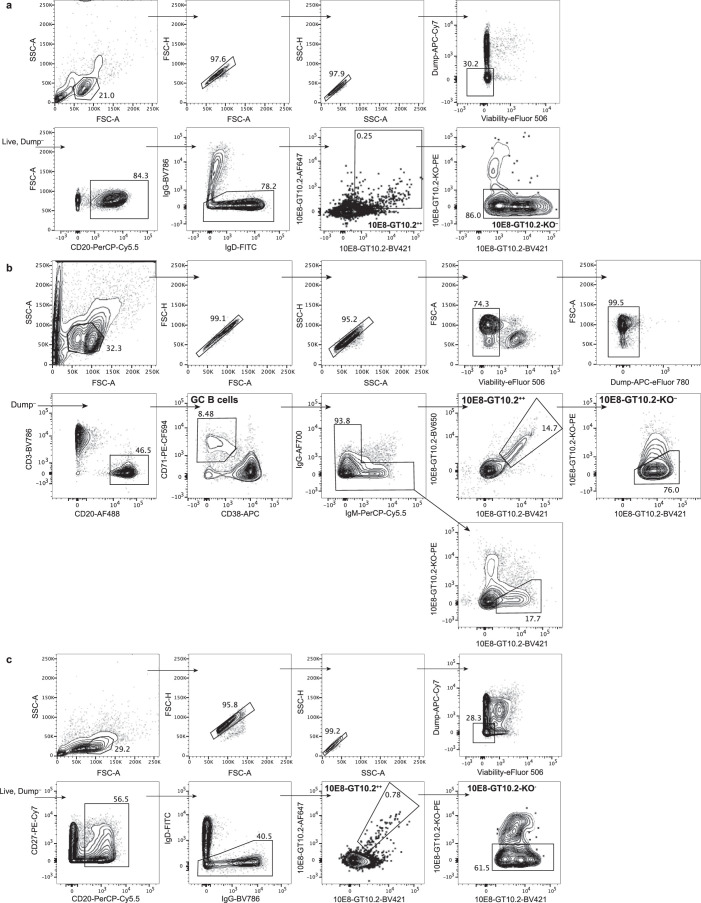
Gating strategy used to assess immune responses to 10E8-GT immunogens in NHPs. **a**, Gating scheme for 10E8-GT10.2-specific (10E8-GT10.2^++^) and 10E8-GT10.2 epitope-specific (10E8-GT10.2^++^10E8-GT10.2-KO^–^) naïve B cells. **b**, Gating scheme for 10E8-GT10.2-specific and 10E8-GT10.2 epitope-specific GC B cells. **c**, Gating scheme for 10E8-GT10.2-specific and 10E8-GT10.2 epitope-specific PBMC-memory B cells.

Two of five known homologs of human D_H_3-3 in Indian rhesus macaques (D_H_3-41*01_S8240 and D_H_3-41*01_S4389) encode the YxFW motif, whereas the remaining alleles encode YxIW (Fig. 7a)^41^, permitting testing of 10E8-GT immunogens in some rhesus macaques. BCR sequencing of sorted 10E8-GT10.2 epitope-specific naive CD20^+^IgG^−^IgD^+^ B cells from unimmunized rhesus macaques (Extended Data Fig. 9a) indicated that sorted BCRs were enriched for long HCDR3s (Fig. 7b). BCRs with 10E8-class HCDR3s (length of 21–24 aa with a YxFW motif at the equivalent position within the HCDR3 as 10E8) were detected in eight of nine macaques with a median frequency of 0.0078% among naive B cells (Fig. 7c), 18-fold lower than their frequency of 0.14% in the human naive B cell repertoire, which made rhesus macaques a viable, although challenging, model to assess 10E8-GT immunogens.

**Extended Data Fig. 10:**
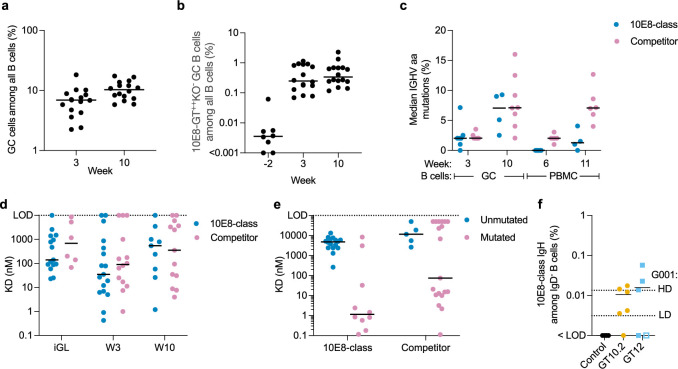
Immunogenicity of 10E8-GT NPs in rhesus macaques. **a**, Percentage of GC B cells (CD38^−^CD71^+^) among all B cells (CD3^−^CD20^+^) in fine needle aspirate samples of rhesus macaques 3 or 10 weeks post immunization with 10E8-GT10.2 12mer, as described in Fig. 7. Each symbol represents an independent lymph node (n = 16 from n = 8 independent animals). **b**, Frequency of epitope-specific (10E8-GT10.2^++^10E8-GT10.2-KO^−^) GC B cells (CD38^−^CD71^+^) observed as a percentage of total B cells (CD3^−^CD20^+^) 2 weeks prior to or 3 or 10 weeks post immunization with 10E8-GT10.2 12mer, as described in Fig. 7. Each symbol represents an independent lymph node (n = 8 for week −2, n = 16 for week 3 and 10). **c**, Median percent amino acid (aa) mutations in the V_H_ of epitope-specific (10E8-GT10.2^++^10E8-GT10.2-KO^−^or 10E8-GT12^++^10E8-GT12-KO^−^) BCRs sorted from macaques immunized with 10E8^−^GT10.2 12mer or 10E8-GT12 12mer at the indicated time-points from GC B cells (CD38^−^CD71^+^) or PBMC-memory B cells (CD20^+^IgD^−^) among BCRs with 10E8-class HCDR3s (10E8-class) compared to BCRs lacking the YxFW motif (Competitor). Symbols represent individual animals, bars indicate median values. **d**, SPR-measured monovalent K_D_ values for 10E8-GT10.2 binding to selected antibodies with 10E8-class HCDR3s (10E8-class) or lacking the YxFW motif (Competitor) induced by 10E8-GT10.2 12mer isolated from the GCs at week 3 (W3) or 10 (W10) post immunization, compared to available matching inferred-germline (iGL) antibodies. Each symbol represents one antibody. **e**, SPR-measured monovalent K_D_ values for 10E8-GT10.2 binding to selected mutated or unmutated antibodies with 10E8-class HCDR3s (10E8-class) or lacking the YxFW motif (Competitor) induced by 10E8-GT10.2 12mer isolated from PBMC-memory (CD20^+^IgD^−^). **f**, Frequency of 10E8-class IgH among PBMC-memory B cells (CD20^+^IgD^−^) at week 11 (GT10.2, n = 6) or week 10 (GT12, n = 5) post immunization compared to macaques immunized with stabilized soluble HIV envelope protein (control, week 10, n = 4). For comparison, dotted lines indicate median frequencies of VRC01-class precursors 8 weeks after high dose (HD) or low dose (LD) immunization with eOD-GT8 60mer in the IAVI G001 human phase 1 clinical trial^5^, with the caveat that frequencies in G001 were measured among IgG^+^ B cells rather than IgD^−^B cells. Open symbols indicate macaques lacking a permissive D_H_3-41 allele. Lines indicate median values.

We used an escalating dose regimen^42^ to immunize eight rhesus macaques with a total of 100 µg of 10E8-GT10.2 12mer and 750 µg of saponin/monophosphoryl lipid A nanoparticle (SMNP)^43^ adjuvant delivered through seven immunizations of increasing doses over 14 days (Fig. 7d). Control macaques (*n* = 4) were immunized using the same protocol with a stabilized soluble HIV-1 Env trimer that lacked the MPER epitope (BG505 MD39 gp140 (ref. ^9^)). Analysis of fine needle aspirates from inguinal draining lymph nodes showed strong CD38^−^CD71^+^ B cell responses in the GCs that persisted through week 10 (Extended Data Figs. 9b and 10a). The 10E8-GT10.2 12mer induced strong epitope-specific GC responses, with epitope-specific 10E8-GT^+^KO^−^ GC B cells comprising 1:400 to 1:300 of total CD3^−^CD20^+^ B cells at weeks 3 and 10, respectively (median frequencies; Extended Data Fig. 10b). Based on genomic sequencing, two of eight macaques immunized with 10E8-GT10.2 12mer that lacked a permissive D_H_3-41 allele (Supplementary Table 5) were excluded from subsequent memory B cell analysis. All 10E8-GT10.2 12mer-immunized macaques produced detectable 10E8-class HCDR3s in both CD38^−^CD71^+^ GCs and CD20^+^IgD^−^ peripheral blood mononuclear cell (PBMC) memory B cells (Fig. 7e), whereas only a single 10E8-like HCDR3 was detected in >9,000 BCRs from macaques immunized with soluble HIV-1 Env. Amino acid mutations in both 10E8-class and non-10E8-class BCRs increased between weeks 3 and 10 in CD38^−^CD71^+^ GC B cells and between weeks 6 and 11 in CD20^+^IgD^−^ PBMC memory B cells (Extended Data Fig. 10c). Despite the increased amino acid mutation levels, binding affinities of 10E8-class BCRs isolated from GCs for 10E8-GT10.2 did not improve between weeks 3 and 10 (Extended Data Fig. 10d). However, mutated 10E8-class antibodies detected in PBMC CD20^+^IgD^−^ memory B cells had substantially higher affinities than unmutated BCRs (median >4,000-fold; Extended Data Fig. 10e), indicating productive affinity maturation. 10E8-like V_H_ genes were significantly enriched in BCRs with 10E8-class HCDR3s (19% compared to 2–3% in non-10E8-class sequences; Fig. 7f and Supplementary Table 5). These data showed that 10E8-GT10.2 12mer consistently induces diverse 10E8-class precursors in NHPs.

We also immunized six rhesus macaques with a total of 100 µg of 10E8-GT12 12mer and 375 µg of SMNP-QS21 using the same dose escalation strategy (Fig. 7d). Sequencing of epitope-specific PBMC memory CD20^+^IgD^−^ B cells at week 10 revealed that three of the three macaques that carried at least one permissive D_H_ allele and with sufficient sequencing depth showed strong enrichment of 10E8-class HCDR3s, comparable to the 10E8-GT10.2 12mer immunizations (Fig. 7e). Overall, the median frequencies of 10E8-class B cells induced by 10E8-GT10.2 12mer and 10E8-GT12 12mer among class-switched memory B cells were 1:9,500 and 1:6,300, respectively (Extended Data Fig. 10f). Seven of eight macaques immunized with 10E8-GT10.2 12mer and all three macaques immunized with 10E8-GT12 12mer with sufficient BCR sequence recovery and at least one permissive D_H_allele produced detectable LN01-class HCDR3s (Fig. 7g), whereas no LN01-class HCDR3s were detected in macaques immunized with Env.

Attempts to solve cocrystal structures of a nonglycosylated variant of 10E8-GT10.2 in complex with several high-affinity Fabs from 10E8-GT10.2 12mer-immunized macaques yielded one structure in complex with a high-affinity (*K*_d_ = 4.8 nM) Fab at 3.1 Å from a macaque that lacked a permissive D_H_ allele. Despite using a YxIW instead of the YxFW motif at the tip of a 22-aa HCDR3, the interaction between Trp^100b^ and 10E8-GT10.2 closely resembled the interaction between mature 10E8 Trp^100b^ and MPER peptide, and the macaque antibody engaged the scaffold at an angle similar to that of mature 10E8 with MPER peptide, although slightly rotated (Fig. 7h), suggesting that even with the D_H_3-41*01 allele, macaque 10E8-GT10.2 12mer-induced antibodies mimicked the 10E8–MPER interaction. As such, 10E8-GT nanoparticles induced 10E8-class bnAb precursors that shared key structural features with mature 10E8 in rhesus macaques.

### Induced BCRs acquire affinity for a boosting candidate

**Fig. 8:**
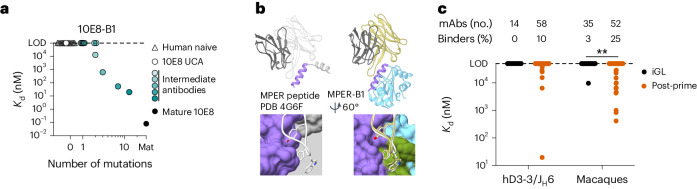
10E8-class BCRs induced by 10E8-GT nanoparticles bind epitope scaffold 10E8-B1 containing a near-native 10E8 peptide epitope. **a**, SPR-measured monovalent *K*_d_ values for 10E8-B1 binding to different antibodies containing the indicated number of 10E8-class mutations, including the 10E8 UCA, 10E8-class human naive precursors isolated by human B cell sorting (human naive), artificial partially mature 10E8-class antibodies (intermediates) and mature 10E8. Each symbol represents an antibody; overlapping data are staggered along the *x* axis. **b**, Crystal structure of 10E8-B1 in complex with 10E8 bnAb (right), with the structure of 10E8 bound to peptide^12^ (left) shown for reference. Colors are as in Fig. 3. **c**, SPR-measured monovalent *K*_d_ values for 10E8-B1 binding to iGL antibodies or antibodies recovered after immunization of hD3-3/J_H_6 mice or macaques with 10E8-GT nanoparticles (after priming). Each symbol represents a different antibody; **P = 0.004. Data were analyzed by a Kruskal–Wallis test with a Dunn’s multiple comparison correction.

Germline-targeting priming immunogens should consistently induce bnAb-precursor memory and/or GC B cells susceptible to boosting by immunogens more similar to the native viral protein (native-like) than the priming immunogen^11^. 10E8-class bnAb precursors induced by 10E8-GT nanoparticles in either hD3-3/J_H_6 mice or NHPs had no neutralizing activity against HIV pseudoviruses (Supplementary Table 6), which was expected because the 10E8 epitope on the priming immunogens was substantially modified from wild-type and lacked steric constraints imposed by the membrane and ectodomain of HIV Env. To determine whether 10E8-GT nanoparticle immunization selected for 10E8-class BCRs that bound to more native-like immunogens, we tested binding of post-prime antibodies to an epitope scaffold (10E8-B1) with a 10E8 peptide epitope that was fully native, except for one mutation required for solubility (W680N, Hxb2 numbering; Extended Data Fig. 1h). 10E8-B1 had no detectable affinity for early 10E8 lineage members but had increasing affinity for artificial intermediate 10E8 lineage members with three or more mutations (Supplementary Table 1) and ultrahigh affinity (*K*_d_ = 80 pM) for mature 10E8 (Fig. 8a).

A 2.63-Å-resolution crystal structure of 10E8-B1 in complex with mature 10E8 bnAb showed that the structure of 10E8-B1 agreed well with the original T117 scaffold (0.61 Å bb-r.m.s.d.) and that the interaction between 10E8-B1 and 10E8 closely resembled the interaction between 10E8 and MPER peptide (Fig. 8b and Extended Data Fig. 3), suggesting that 10E8-B1 has appropriate antigenicity and structure to probe for 10E8-like maturation in 10E8-GT-induced antibodies. 10E8-B1 bound to 10% of 10E8-class antibodies induced by 10E8-GT nanoparticles, including 10E8-GT10 12mer or 10E8-GT12 24mer in hD3-3/J_H_6 mice (Fig. 8c and Supplementary Table 2), and to 25% of 10E8-class antibodies primed by 10E8-GT10 12mer in macaques (Fig. 8c). Binding of 10E8-B1 to germline-reverted 10E8-class antibodies from macaques was significantly weaker than to the matching antibodies primed by 10E8-GT10 12mer in macaques (Fig. 8c), indicating that binding by 10E8-B1 was due to SHM acquired by these antibodies. Thus, 10E8-GT nanoparticle immunization selects for affinity maturation that confers affinity for an antigen with a more native-like 10E8 epitope.

## Discussion

By combining germline targeting with epitope scaffolding and nanoparticle design, we developed immunogens that consistently induced 10E8-class HIV bnAb precursors with bnAb-associated genetic and structural features, including long HCDR3s with specific binding motifs that confer the potential to develop into bnAbs, in rhesus macaques and two mouse models. We also showed that the priming immunogens selected for productive directional affinity maturation, such that at least a subset of the induced bnAb precursors had affinity for a more native-like antigen. These findings provide proof of principle that epitope scaffolds can be designed to induce responses from rare, HCDR3-dominant bnAb precursors and select for a degree of favorable maturation in those precursors, extending the functionality of the epitope scaffold approach^31,44–47^.

Development of B cells expressing precursors for MPER bnAbs 2F5 and 4E10 was reported to be blocked by tolerance barriers^21–24^. We found normal B cell development for 10E8-class precursors. The 10E8-GT epitope scaffolds also induced precursors for a related yet genetically distinct class of bnAb, LN01, demonstrating the capacity for multi-bnAb precursor priming without obvious interference from tolerance mechanisms, consistent with the low poly- or autoreactivity exhibited by 10E8- and LN01-class lineages^12,13^.

Our observations validated 10E8-GT nanoparticle germline-targeting priming immunogens for consistent induction of diverse bnAb precursors across vaccinated mice and NHPs. Germline-targeting vaccine design posits that bnAbs can be elicited by first priming bnAb precursors with the necessary bnAb-associated genetic and structural features and then using a series of boosters of increasing similarity to the native glycoprotein to select for the necessary SHM to produce bnAbs. Hence, additional work is needed to develop sequential heterologous boosting regimens to induce 10E8-class bnAbs. We envision boosting immunogens to include an epitope scaffold nanoparticle with a more native-like MPER epitope such as 10E8-B1, followed by one or more membrane-bound envelope protein(s) to select for maturation to enable 10E8-like and LN01-like BCRs to engage the native MPER peptide and its surroundings on the membrane-anchored Env glycoprotein.

Based on these data, we confirm the MPER as an attractive HIV vaccine target and propose 10E8-GT nanoparticles as MPER vaccine priming immunogens. Our epitope scaffolds bound to and isolated human naive bnAb precursors from human PBMCs, suggesting that the positive immunization data from mice and macaques have the potential for translation to humans. Our finding that 10E8-GT12 24mer delivered by mRNA-LNP induces similar 10E8-class B cell responses as SMNP-adjuvanted protein immunization in a stringent mouse model supports the potential for rapid clinical testing. The data further encourage the development of germline-targeting epitope scaffold nanoparticles to induce bnAb precursors and initiate bnAb induction for other epitopes that are sterically occluded or poorly immunogenic in the context of native viral glycoproteins, such as the MPER of Filoviridae^48^, the influenza A hemagglutinin anchor^3^ or the relatively conserved S2 subunit in betacoronaviruses^4^.

## Methods

This work complies with all relevant ethical regulations. Animal experiments were approved by the Institutional Animal Care and Use Committees (IACUCs) of Harvard University, Massachusetts General Hospital (MGH), Alpha Genesis and Emory University. Experiments involving human samples were approved by the La Jolla Institute for Immunology (LJI) Institutional Review Board.

### Human immunoglobulin repertoire bioinformatic analysis

MPER bnAb-precursor frequencies were estimated from publicly available NGS data of ~1.1 × 10^9^ heavy chain sequences from 14 HIV-seronegative donors, as previously described^7,51^. Datasets were searched for sequences matching the following filters: 10E8-class HCDR: length 21–24 + regex ‘Y.FW’ starting at position 7 for lengths 21–22 or position 8 for lengths 23–24; 10E8-class IgH: 10E8-class HCDR3 + V_H_3-15, V_H_3-49, V_H_3-72 or V_H_3-73; LN01-class HCDR3: length 20–23 + regex ‘…….FW…[YF].[WFY]….’ for length 20, regex ‘…….FW…[YF].[WFY]….’ for length 21–22 or regex ‘………FW…[YF].[WFY]….’ for length 23; LN01-class IgH: LN01-class HCDR + V_H_4-39 or V_H_4-59. Precursor frequencies were estimated by dividing the total number of precursors matching the respective definition by the sum of sequences for the individual.

### Immunogen design

Visual inspection of Protein Data Bank (PDB) IDs 4G6F and 5T85 suggested a clash between K52 of 10E8-iGL1 (Kabat numbering) and F673 of the MPER (HxB2 numbering, F121 of T117). Computational modeling using the Rosetta Software suite^52,53^ resulted in 10E8-GT2 that resolved this clash and bound weakly (1.5 µM) to 10E8-iGL1. Further Rosetta fixbb design yielded GT3 and GT4, with improving affinities for 10E8-iGL1. Next, directed evolution by yeast surface display^34,54^ was used to select for variants capable of binding to increasingly more germline-reverted variants of 10E8. Selection with 10E8-iGL1 led to 10E8-GT5 that bound to 10E8-iGL1 with 81 nM affinity. Next, we divided the epitope on 10E8-GT5 into surface patches of three or four residues and performed ‘combinatorial NNK patch scanning’ by yeast display. In contrast to traditional deep scanning mutagenesis, which only analyzes the effect of single point mutations, these combinatorial NNK patches contained all combinations of all 21 aa (including the stop codon) at the respective positions, thereby allowing potential compensatory mutations to occur. By analyzing four different patches in parallel (residues 71–74, 93–96, 111–114 and 114–115 + 117–118 of T117, respectively), most contact residues were optimized. Each combinatorial NNK patch was enriched against antibodies 10E8-iGL1, 10E8-iGL2 and 10E8-iGL3. The best overall resulting variant, 10E8-GT7, resulted from patch 3 and bound 10E8-iGL1 with 8.2 nM affinity.

One design goal was the multimerization of the immunogen into self-assembling nanoparticles displaying the germline-targeting epitope scaffold. However, the T117v2 scaffold was not well suited for this goal because both N and C termini of the scaffold are near the epitope; hence, genetic fusion of the epitope scaffold to a self-assembling protein would result in poor exposure of the epitope on the nanoparticle. With generation 10E8-GT8, we switched from T117v2 to T298, a previously described circularly permuted variant of T117 with N and C termini opposite the epitope^32^. However, the original T298 suffered from low expression levels and relatively low thermal stability (48 °C), and it dimerized in solution^32^. Resurfacing of T298 using the dTERMen algorithm^55^ was used to improve stability and solubility of the scaffold. We genetically fused a circularly permuted immunogen, 10E8-GT8, to several self-assembling nanoparticle platforms and managed to purify high yields (~24 mg l^–1^) of fully assembled particles after fusion to a glycosylated variant of the dodecameric 3-dehydroquinase from *T. thermophilus*.

Another key design goal was the introduction of N-linked glycosylation sites onto the scaffold to focus B cell responses on the MPER epitope. We initially explored the introduction of single artificial N-linked glycosylation sites into irrelevant surfaces of the epitope scaffold of 10E8-GT8.1. Sites that decreased affinity for 10E8-iGL2 by no more than 1.3-fold and decreased expression yields by no more than 40% were selected for further investigation. We next tested combinations of multiple glycosylation sites on 10E8-GT8.1 and obtained 10E8-GT8.2 with four N-linked glycosylation sites. We further increased the number of N-linked glycosylation sites with each subsequent generation of immunogens.

Although 10E8-GT8.2 showed strong binding to 10E8-iGL1 (*K*_d_ = 13 nM), it did not bind the 10E8 UCA nor any of the 55 NGS-derived precursors tested, all of which differ from 10E8-iGL1 in their respective HCDR3s but are otherwise identical (Supplementary Tables 1 and 2). The key residues of 10E8 are the D_H_-encoded residues at the tip of the HCDR3, which were present in all 10E8-class precursors used in this study. We hypothesized that additional interactions of the scaffold with these key residues might increase the affinity and breadth for precursors that share these features. At this stage, we had not yet taken full advantage of the combinatorial NNK patch scanning yeast display optimization of 10E8-GT5 performed earlier, because 10E8-GT7 and 10E8-GT8 only contained mutations from patch 3, ignoring results from the other patches. Patch 2 had analyzed residues 93–96 (equivalent to 75–78 in circularly permutated T298), which form a loop of the scaffold in close proximity to the D gene-encoded ‘FW’ motif of bound 10E8. Enrichment of this library with 10E8-iGL3 had resulted in complete redesign of the 75–78 loop with the final sequence ‘GWYQ’, which we hypothesized to form a pocket that would result in additional beneficial contacts with the D_H_-encoded key residues.

We combined this new D_H_ binding loop into a combinatorial yeast library with other promising residues from the remaining combinatorial NNK patch screenings and enriched for binding to 10E8-class precursors. Enrichment with 10E8 UCA resulted in 10E8-GT9.1 that bound to the 10E8 UCA and several NGS precursors. Reversion of each MPER mutation revealed that mutation N132I (N677I in Hxb2 numbering) had no impact on binding affinity; hence, this mutation was subsequently removed. To improve expression and solubility, we manually inspected models of T298-GT9.1 and reverted exposed hydrophobic residues to the respective amino acids in the original T298 before resurfacing. By combining these changes with further refinement of the glycan shield, we obtained 10E8-GT9.2, which bound weakly to the 10E8 UCA and several NGS precursors (21 of 33 tested precursors; Fig. 2a). Further optimization of the D_H_ gene binding pocket by yeast display of an error-prone PCR library (Genemorph II Random Mutagenesis kit, Agilent) added mutation P41Q, resulting in 10E8-GT10.1, which bound weakly to 28 of 33 10E8-like precursors and had modest affinity for the 10E8 UCA (*K*_d_ of ~600 nM). However, nanoparticle constructs presenting 10E8-GT10.1, created by fusion to several self-assembling proteins, failed to express in 293F cells. We therefore further refined the scaffold for increased expression by removing exposed hydrophobic patches, adding additional glycosylation sites and further improving the D binding pocket by incorporating mutation V42A, discovered from yeast display of an error-prone PCR library of GT10.1 that was simultaneously enriched with 10E8 UCA Fab (directly conjugated to Alexa Fluor 647 (AF647)) and NGS-57 (stained with PE-conjugated anti-human Fcγ secondary antibody; Jackson ImmunoResearch). Fusion of the resulting protein, termed 10E8-GT10.2, to the glycosylated 3-dehydroquinase from *T. thermophilus* via a linker that incorporated the PADRE^56^ epitope yielded homogenous particles of the expected molecular weight, termed 10E8-GT10.2 12mer (of note, unexpectedly, the addition of PADRE to the linker in the GT10.2 12mer increased expression levels substantially).

Although 10E8-GT9 and 10E8-GT10 immunogens bound to several NGS precursors, they bound much more weakly to mature and artificial intermediate 10E8 lineage members than to previous immunogen generations. We therefore transferred the MPER of 10E8-GT8, the most advanced version that retained strong binding to mature and intermediate 10E8 variants, onto 10E8-GT10.2, moved an N-linked glycosylation site into the scaffold surface patch engaged by 10E8-NGS-03 and added mutation W680N to the MPER graft. The resulting construct, termed 10E8-GT11, bound with high affinity to mature 10E8 (*K*_d_ = 1.4 nM) but interacted only weakly with 10E8-class precursors. Additional optimization by yeast display was performed by identifying beneficial mutations using an error-prone PCR library, followed by screening of a combinatorial library that combined identified mutations using NGS precursors. The resulting epitope scaffold, termed 10E8-GT12, engaged 46% of 10E8-class precursors tested with affinities comparable to GT10 while binding strongly (*K*_d_ = 1.0 nM) to mature 10E8 (Fig. 2). We fused 10E8-GT12 to the same PADRE-containing 12mer nanoparticle platform described above and obtained well-formed particles, termed 10E8-GT12 12mer (Extended Data Fig. 1d). We also created 10E8-GT12 24mer by fusing 10E8-GT12 to each terminus of the 3-dehydroquinase nanoparticle protomers. In the 10E8-GT12 24mer, rather than using linkers with PADRE, we included exogeneous T-help peptides derived from *Aquifex aeolicus* lumazine synthase that were found to be broadly immunogenic in humans^57^.

We also developed antigens with more native 10E8 epitope grafts to serve as candidate boost immunogens to follow the prime and to serve as tools to probe the maturation of 10E8-class antibodies induced by the prime. To reduce binding of irrelevant antibodies, we resurfaced T298 using the dTERMen algorithm^55^, and we eliminated remaining hydrophobic surface patches manually. Similar to the original T117 (ref. ^31^) and T298 (ref. ^32^) scaffolds, many designs formed dimers or aggregates in solution. Inspection of the previously published crystal structure of T298 (PDB ID 3T43) led to inclusion of bulky residues at positions 55 (methionine) and 77 (N-linked glycosylation site) to disrupt dimer formation without altering 10E8 bnAb binding. We also added the D binding pocket from GT12 to maintain the critical interaction with the FW motif within the HCDR3s of 10E8-class antibodies. We removed N-linked glycosylation sequons that we had found to be unoccupied in 10E8-GT12. We fused a series of such candidate epitope scaffolds to the same glycosylated 3-dehydroquinase nanoparticle described above, either at the nanoparticle C terminus (12mer) or at both N and C termini (24mer). Incorporation of a consensus 10E8 epitope graft led to aggregation, but we obtained homogenous nanoparticles by including a single germline-targeting mutation (W104N; W680N in Hxb2 numbering). This nanoparticle was termed 10E8-B1 24mer, and the corresponding monomeric epitope scaffold was termed 10E8-B1.

### Protein expression, purification, biotinylation and biochemical characterization

Genes of proteins and antibodies were synthesized and cloned into pHLSec or its variant pCWSec by Genscript using codons optimized for expression in human cells. Proteins were expressed and purified as described in detail previously^51^. Briefly, plasmids were transfected into FreeStyle 293F cells (Thermo Scientific), and expression was performed in protein-free chemically defined FreeStyle medium (Thermo Scientific). His-tagged proteins were purified from clarified supernatants using immobilized metal affinity chromatography followed by size-exclusion chromatography (SEC), nanoparticles were purified using *Galanthus nivalis* lectin affinity chromatography (Vector Laboratories) followed by SEC, and antibodies were purified by protein A affinity chromatography followed by buffer exchange into Tris-buffered saline. High-throughput expression of antibodies was performed in 96-well plates using the ExpiCHO system (Thermo Scientific) and purified by protein A affinity purification as previously described^38^. Sorting probes were expressed with a C-terminal AviHis tag (GSGGSGLNDIFEAQKIEWHEGSGGHHHHHH**, where ‘*’ denotes a stop codon) and purified by metal affinity chromatography and SEC, as described above. Matching KO probes for each immunogen were generated that incorporated five KO mutations (672A, 673R, 675R, 680E and 683D; Hxb2 numbering) in the MPER. Purified proteins were biotinylated by BirA enzymatic reaction (Avidity) according to the manufacturer’s protocol and purified by SEC. Immunogen candidates were characterized by SEC coupled with multiangle light scattering on a Dawn 18 instrument (Wyatt Labs) and Optilab dRI detector using ASTRA 7.1.1.3 software, as previously described^51^. Protein stability was determined by dynamic scanning calorimetry (DSC) on a MicroCal VP-Capillary DSC (Malvern Instruments) as described previously^51^.

### SPR

All *K*_d_ values for antibody–antigen interactions presented in main text figures were measured on a Carterra LSA instrument using HC30M or CMDP sensor chips (Carterra) and 1× HBS-EP+ (pH 7.4) running buffer (20× stock from Teknova, H8022) supplemented with bovine serum albumin at 1 mg ml^–1^. Carterra Navigator software instructions were followed to prepare chip surfaces for ligand capture. In a typical experiment, approximately 2,500 to 2,700 RU of capture antibody (SouthernBiotech, 2047-01) at 25 µg ml^–1^ in 10 mM sodium acetate (pH 4.5) was amine coupled using a commercial Amine Coupling kit (GE, BR-1000-50) but using tenfold diluted NHS and EDC concentrations. Regeneration solution was 1.7% phosphoric acid injected three times for 60 s per each cycle. The solution concentration of ligands was around 1 µg ml^–1^, and contact time was 5 min. Raw sensograms were analyzed using Carterra Kinetics software (Carterra), interspot and blank double referencing, Langmuir model. For fast off-rates (>0.009 s^–1^), we used automated batch referencing that included overlay *y* aline and higher analyte concentrations. For slow off-rates (≤0.009 s^–1^), we used manual process referencing that included serial *y* aline and lower analyte concentrations. After automated data analysis by Kinetics software, a custom R script was used to remove datasets with maximum response signals smaller than signals from negative controls.

Some of the *K*_d_ values in supplementary tables were determined on a ProteOn XPR36 (Bio-Rad) using a GLC Sensor Chip (Bio-Rad) and ProteOn Manager software or Biacore 4000 with CM5 Series S Sensor Chips, as described previously^51^. The same analyte–ligand pair would produce similar *K*_d_ values on all systems tested within a factor of two.

### Site-specific glycosylation profiling

Two methods were used to analyze glycosylation profiles: single-site glycan profiling and DeGlyPHER.

Single-site glycan profiling was performed as described previously^58^. Briefly, proteins were denatured, reduced and alkylated, followed by enzymatic digestion using trypsin, chymotrypsin or α-lytic protease. Peptides were analyzed by nanoLC-ESI mass spectrometry with an UltiMate 3000 HPLC (Thermo Fisher Scientific) system coupled to an Orbitrap Eclipse mass spectrometer (Thermo Fisher Scientific). Peptides were separated using an EASY-Spray PepMap RSLC C18 column (75 μm × 75 cm) with an in-line trapping column (PepMap 100 C18 3 μM, 75 μM × 2 cm). Data were analyzed using protein metrics Byos software (version 3.5). The relative amounts of each glycan at each site as well as the unoccupied proportion were determined by comparing the extracted ion chromatographic areas for different glycopeptides to an identical peptide sequence. Glycans were categorized according to the composition detected.

DeGlyPHER was performed as described previously^59^. Briefly, proteins were deglycosylated with Endo H, digested with proteinase K and deglycosylated again with Endo H, followed by lyophilization and resupension in PNGase F-containing H_2_^18^O. Samples were analyzed on a Q Exactive HF-X mass spectrometer. Protein and peptide identification were performed using the Integrated Proteomics Pipeline (IP2) using the automated GlycoMSQuant (Baboo et al.^59^) implementation. GlycoMSQuant summed precursor peak areas across replicates, discarded peptides without NGS, discarded misidentified peptides when N-glycan remnant mass modifications were localized to non-NGS asparagines and corrected/fixed N-glycan mislocalization where appropriate.

### Structure determination by crystallography

10E8-GT4–10E8 iGL, 10E8-GT10.1–NGS precursor and 10E8-GT11–10E8-iGL1 complexes were adjusted to 8 to 10 mg ml^–1^ in 100 mM HEPES and 150 mM NaCl (pH 7.4) buffer. Purified 10E8-GT10.2–NHP W3-01 and 10E8-B1–mature 10E8 complexes were adjusted to 10 mg ml^–1^ in TBS buffer (20 mM Tris and 150 mM NaCl), pH 7.6 and 7.4, respectively. The complexes were screened for crystallization on an HTP robotic CrystalMation system (Rigaku) against the 384 conditions of the JCSG 1-4 Core Suite (NeXtal; Rigaku Reagents) in sitting drop format with 0.1 μl of protein and 0.1 μl of reservoir solution. Crystals were collected, soaked in reservoir solution containing the respective cryoprotectant listed below, flash cooled and stored in liquid nitrogen until data collection. 10E8-GT4–10E8 iGL crystals grew in 10% glycerol, 0.1 M HEPES (pH 7.5) and 5% PEG 3000 with 26% (vol/vol) glycerol as cryoprotectant. 10E8-GT10.1–NGS precursor crystals grew in 0.1 M imidazole (pH 8) and 40% PEG400 with 40% (vol/vol) PEG 400 acting as cryoprotectant. 10E8-GT11–10E8-iGL1 crystals grew in 0.095 M sodium citrate, 19% 2-propanol, 5% glycerol and 19% PEG4000 with 26% (vol/vol) glycerol as cryoprotectant. 10E8-GT10.2–NHP W3-01 crystals grew in 0.2 M ammonium dihydrogen phosphate and 20% PEG3350 with 10% (vol/vol) ethylene glycol as cryoprotectant. 10E8-B1–mature 10E8 crystals grew in 0.2 M calcium chloride and 20% PEG3350 with 20% (vol/vol) glycerol as cryoprotectant. Diffraction data were collected at cryogenic temperature (100 K) at the respective synchrotron beamlines indicated in Extended Data Fig. 3. The diffraction data were processed with HKL2000 (ref. ^60^). The 10E8-GT4–10E8 iGL, 10E8-GT10.1–NGS precursor and 10E8-GT11–10E8-iGL1 complex structures were solved by molecular replacement with Phaser^61^ using the 10E8 Fab and T117v2 scaffold structures from PDB 5T6L as search models. For the 10E8-GT10.2–NHP W3-01 complex, the scaffold structure from PDB 5T80 and a V_H_–V_L_ model generated by Repertoire Builder (https://sysimm.org/rep_builder/) for the NHP W3-01 Fab were used. The 10E8-B1–mature 10E8 complex structure was subsequently determined by molecular replacement with Phaser using the 10E8-iGL1 Fab and 10E8-GT11 scaffold structures from the 10E8-GT11–10E8-iGL1 complex structure as a search model. Iterative model building and refinement were performed in Coot^62^ and PHENIX^63^, respectively, and Ramachandran statistics were validated in MolProbity^64^.

### Structure determination by cryo-EM

10E8-GT10.2 (120 μg) was incubated with the on-target mature 10E8 Fab (300 μg) and an off-target W6-10 Fab (300 μg) in an equal molar ratio (1:1:1) overnight at room temperature. The complex was then purified over a Superdex 200 Increase column (GE Healthcare) and concentrated to 2.5 mg ml^–1^. Next, 3 μl of the complex was mixed with 0.5 μl of 35 μM lauryl maltose neopentyl glycol (Anatrace; final concentration of 5 µM) before deposition onto 1.2/1.3 UltrAuFoil 200 grids (EMS; glow-discharged for 10 s), directly preceding vitrification using a Vitrobot Mark IV (Thermo Fisher Scientific) with the following settings: 4 °C, 100% humidity, 10-s wait time, 6-s blot time and blot force of 2. Once the sample was deposited, the grids were blotted and plunged into liquid ethane to immobilize the particles in vitreous ice. Movie frames were collected using EPU image acquisition software (Thermo Fisher Scientific) at a nominal magnification of ×190,000 with a Thermo Fisher Scientific Falcon 4 detector mounted on a Thermo Fisher Scientific Glacios operating at 200 kV. Counting mode was used, with a total exposure dose of 53 e^–^ Å^–2^. In total, 4,249 micrographs were motion, dose and CTF corrected using cryoSPARC Live imported into cryoSPARC^65^ (Extended Data Fig. 3). Template Picker was used to pick 956,668 particles, which were then extracted and two-dimensionally classified. The particles in selected two-dimensional classes were further filtered by ab initio reconstruction using C1 symmetry, resulting in 56,628 particles subjected to nonuniform refinement. The final reconstruction was estimated at ~4.0-Å resolution using Fourier shell correlation and a 0.143 cutoff (Extended Data Fig. 3).

For model building, an initial model of 10E8 Fab and MPER scaffold was generated using PDB 5T85 and docked into the cryo-EM map using UCSF ChimeraX^66^. Coot 0.9.8 (ref. ^67^), Phenix^68^ and Rosetta^52,53^ were used for model building and refinement (Extended Data Fig. 3). The final model and map have been deposited in the PDB and Electron Microscopy Data Bank under accession codes 8SX3 and EMD-40825, respectively.

### Human B cell repertoire screening and sorting

Leukoreduction (LRS) tubes from healthy donor samples were obtained from the San Diego Blood Bank from consenting participants, in accordance with protocols approved by the LJI Institutional Review Board. PBMCs were isolated from blood by the LJI Blood Processing Core and were frozen and stored in liquid nitrogen until analysis.

Cryopreserved PBMCs were thawed and recovered in RPMI medium containing 10% fetal bovine serum (FBS) supplemented with 1× penicillin/streptomycin (pen/strep) and 1× GlutaMAX (R10). Fluorescently labeled antigen probes were prepared by mixing fluorophore-conjugated streptavidin with small volumes of biotinylated antigen probes in 1× PBS at room temperature, with additions every 15 to 20 min for a total of 45 min to 1 h depending on the human naive B cell screening experiment.

For 10E8-GT9.2 and 10E8-GT10.1 human naive B cell screening using direct lysis sorts and single-cell BCR amplification, cells were first stained with either 10E8-GT9.2-KO or 10E8-GT10.1-KO probe and then the respective wild-type probes for a total of 45 min. Without washing, the cells were then stained with the antibody master mix for an additional 30 min. Cells were then washed twice in R10 and sorted on a FACSAria II (BD Biosciences).

For the 10E8-GT10.1 human naive B cell screening using 10x Genomics single-cell BCR sequencing, anti-AF647 antigen-specific B cell enrichment was performed. Cells were first incubated with 10E8-GT10.1-AF647 probe in R10 medium for 60 min at room temperature and then washed with 1% bovine serum albumin in PBS. The cells were then incubated with anti-Cy5/anti-AF647 microbeads for isolation of 10E8-GT10.1-AF647 probe-binding cells, following the user guide provided (Miltenyi Biotec). The purified cells were counted and stained with a mix of tetramer probes (the other 10E8-GT10.1 probe and a 10E8-GT10.1-KO probe). Without washing, the antibody master mix was added to the cells for another 30 min. Anti-human TotalSeq-C hashtag antibodies (BioLegend) were also added at this time at a concentration of 0.1 µg per 1 million cells. Cells were then washed twice in R10 before sorting on a FACSAria II (BD Biosciences).

For 10E8-GT12 human naive B cell screening, total B cells were enriched by negative selection using an EasySep human B cell isolation kit (StemCell). Purified B cells were then counted and incubated with the fluorescently labeled antigen probes. First, 10E8-GT12-KO probe was added for 15 min at 4 °C and then incubated with wild-type 10E8-GT12 probes for an additional 15 min. Without washing, Fc Block (BD Biosciences) was added for 5 min and stained with surface antibodies for an additional 30 min at 4 °C. Cells were washed twice with FACS buffer (PBS + 2% FBS + 1 mM EDTA) and sorted on a FACSymphony S6 (BD Biosciences).

The following reagents were used for staining in the human naive B cell screening experiments:

1. 10E8-GT9.2: AF647 streptavidin (Invitrogen), BV421 streptavidin (BioLegend), PhycoLink streptavidin-RPE (ProZyme), BB515 streptavidin (BD Biosciences), mouse anti-human CD19 PE-Cy7 (HIB19, Thermo Fisher Scientific), mouse anti-human CD3 APC-eFluor 780 (UCHT1, Thermo Fisher Scientific), mouse anti-human CD14 APC-eFluor 780 (61D3, Thermo Fisher Scientific), mouse anti-human CD16 APC-eFluor 780 (eBioCB16, Thermo Fisher Scientific), mouse anti-human IgG APC-Cy7 (HP6017, BioLegend) and eBioscience Fixable Viability Dye eFluor 780 (Invitrogen).
2. 10E8-GT10.1 (single-cell BCR amplification): AF647 streptavidin (Invitrogen), BV421 streptavidin (BioLegend), PhycoLink streptavidin-RPE (ProZyme), mouse anti-human CD19 PE-Cy7 (HIB19, Thermo Fisher Scientific), mouse anti-human CD3 APC-eFluor 780 (UCHT1, Thermo Fisher Scientific), mouse anti-human CD14 APC-eFluor 780 (61D3, Thermo Fisher Scientific), mouse anti-human CD16 APC-eFluor 780 (eBioCB16, Thermo Fisher Scientific), mouse anti-human IgG APC-Cy7 (HP6017, BioLegend) and eBioscience Fixable Viability Dye eFluor 780 (Invitrogen).
3. 10E8-GT10.1 (10x Genomics): AF647 streptavidin (Invitrogen), BV421 streptavidin (BioLegend), PhycoLink streptavidin-RPE (ProZyme), mouse anti-human CD19 PE-Cy7 (HIB19, Thermo Fisher Scientific), mouse anti-human CD3 APC-eFluor 780 (UCHT1, Thermo Fisher Scientific), mouse anti-human CD14 APC-eFluor 780 (61D3, Thermo Fisher Scientific), mouse anti-human CD16 APC-eFluor 780 (eBioCB16, Thermo Fisher Scientific), mouse anti-human IgG APC-Cy7 (HP6017, BioLegend), propidium iodide (Thermo Fisher Scientific) and TotalSeq-C anti-human Hashtag antibody 5 (LNH-94 and 2M2, BioLegend).
4. 10E8-GT12: AF647 streptavidin (BioLegend), BV421 streptavidin (BioLegend), PE-streptavidin (BioLegend), mouse anti-human CD3 APC-Cy7 (UCHT1, BioLegend), mouse anti-human CD14 APC-Cy7 (M5E2, BioLegend), mouse anti-human CD16 APC-Cy7 (3G8, BioLegend), mouse anti-human CD20 PE-Cy7 (2H7, BioLegend), mouse anti-human IgG BV605 (G18-145, BD Biosciences), mouse anti-human IgD BUV395 (IA6-2, BD Biosciences), mouse anti-human CD27 BB515 (M-T271, BD Biosciences) and eBioscience Fixable Viability Dye eFluor 506 (Invitrogen).

### Human BCR sequencing using single-cell BCR amplification

Single-cell BCR amplification was performed similar to previously described protocols^7,34,37^. Briefly, single B cells were sorted directly into 10–20 μl of lysis buffer. Lysed cells were immediately frozen on dry ice and moved to −80 °C for storage. First-strand cDNA synthesis was performed using SuperScript II RT (Invitrogen), following the stated instructions. Heavy and light chain gene transcripts were amplified using a modified nested PCR protocol^69^. For the heavy chain reactions, pooled primers were used at 25 nM, with 50-cycle PCR reactions (polymerase activation, 98 °C for 30 s; denaturation, 98 °C for 15 s; annealing, 62 °C for 20 s; extension, 72 °C for 35 s). For light chain reactions, pooled primers were used at 250 nM, and 25-cycle PCR reactions were used. Phusion Taq polymerase (Thermo Fisher, F530L) was used at 0.5 U per reaction for all reactions. Primers were used from previously published protocols^69^. PCR products were run on 2% agarose E-Gels (Life Technologies). Reactions with 300- to 400-base pair products were sequenced in both directions. Sequencher 5.0 was used to align sequences, and IMGT/V-QUEST was used for V(D)J assignments.

### Human BCR sequencing using 10x Genomics

Sorted cells were prepared for 10x single-cell V(D)J sequencing similar to previously published protocols^70^. For the 10E8-GT10.1 human naive B cell screening, single indexed V(D)J and Feature Barcode libraries were generated following the user guide for the Chromium Single Cell V(D)J Reagent kits with Feature Barcoding technology (Legacy version, 10x Genomics). All libraries were pooled, and sequencing was performed on a NovaSeq Sequencer (Illumina). The V(D)J contigs were assembled and annotated using Cell Ranger v3.0.2 using an immunoglobulin library compiled from IMGT references. The constants.py file was modified to increase the maximum CDR3 length to 110 nucleotides. A Python script was used to associate hashtag read counts with the productive assembled V(D)J sequences, compiling the data into a tabular format^70^.

For 10E8-GT12 human naive B cell screening, V(D)J libraries were prepared using the Dual Indexed 10x Genomics V(D)J 5′ v.2 according to the manufacturer’s protocol (10x Genomics). Raw sequencing data were processed using Cell Ranger v6.1.2. V(D)J contigs were generated and aligned to the prebuilt human reference (refdata-cellranger-vdj-GRCh38-alts-ensembl-7.0.0). V(D)J output was further processed following the Immcantation pipeline^71^. Briefly, contigs were annotated using IgBlast on the IMGT database, and only productive sequences were kept for downstream analysis. Heavy and light chain contigs were paired, and cells with more than one heavy chain sequence were removed. Final analysis was performed using Sequencing Analysis and Data library for Immunoinformatics Exploration (SADIE), and sequences were filtered for IgM^+^ sequences based on the ‘c_call_heavy’ field.

### HEp-2 cell staining assay

The HEp-2 cell staining assay was performed using kits purchased from Aesku Diagnostics, according to the manufacturer’s instructions. These Aesku slides use optimally fixed human epithelial (HEp-2) cells (ATCC) as a substrate and affinity-purified, fluorescein isothiocyanate (FITC)-conjugated goat anti-human IgG for detection. Briefly, 2.5 μg or 25 μl of 100 μg ml^–1^ mAb and controls were added to wells and incubated on HEp-2 slides in a moist chamber at room temperature for 30 min. After incubation, the slides were removed from the incubator chamber and rinsed with PBS buffer. To prevent cross-contamination, a stream of PBS buffer was run along the midline of the slide, allowing the buffer to run off the lower edge of the slide. After the washing procedure, 25 μl of FITC-conjugated goat anti-human IgG was immediately applied to each well, and the slide was returned to the incubator chamber. The slides were allowed to incubate at room temperature in a moist chamber for another 30 min. Subsequently, the slides were washed in the same manner as described above and then mounted on coverslips using the provided mounting medium.

Slides were viewed at 20× magnification and photographed on an EVOS f1 fluorescence microscope at a 250-ms exposure with 100% intensity. Positive- and negative-control sera were provided by the vendor. Samples that demonstrated fluorescence greater than the negative control were considered positive for HEp-2 staining.

### MPER-18 *IgH* KI mouse construction, characterization and immunization studies

MPER-HuGL18^H^ mice were generated following published protocols^39,72^. In brief, the targeting vector 4E10 was modified by the incorporation of human rearranged MPER HuGL18 V(D)J (heavy chain construct) sequences downstream of the promoter region and by elongation of the 5′ and 3′ homology regions using the Gibson assembly method (New England Biolabs). The targeting vector DNA was confirmed by Sanger sequencing (Eton Bioscience). Next, fertilized mouse oocytes were microinjected with a donor plasmid containing the prerearranged MPER HuGL18 *IgH* with the mouse VHJ558 promoter, two pairs of single guide RNAs (25 ng ml^–1^) targeting the H locus and AltR-Cas9 protein (50 ng ml^–1^) and injection buffer^39^. Following culture, resulting zygotes were implanted into the uteri of pseudopregnant surrogate C57BL/6J mothers.

For experiments, male B6.SJL-*Ptprc*^a^*Pepc*^b^/BoyJ mice (CD45.1^+/+^) 8–12 weeks of age were purchased from The Jackson Laboratory. F0 mice from the MPER-HuGL18^H^ mouse (CD45.2^+/+^) colony were bred at the animal facility of the Gene Modification Facility (Harvard University), and breeding for colony expansion and experimental procedures was subsequently performed at the Ragon Institute of Mass General, MIT and Harvard. Ear or tail snips from MPER-HuGL18^H^ mice were genotyped by TaqMan assay under a fee-for-service agreement (TransnetYX). TaqMan probes for the genotyping assay were developed by TransnetYX. CD45.2^+^ B cells from MPER-HuGL18^H^ donor KI mice were enriched using a Pan-B Cell Isolation kit II (Miltenyi Biotec), counted, diluted to the desired cell numbers in PBS and adoptively transferred retro-orbitally into CD45.1^+^ recipient mice, as reported previously^33^. All experiments were performed under the approval of the IACUC of Harvard University and the MGH (animal study protocols 2016N000022 and 2016N000286) and were conducted in accordance with the regulations of the Association for Assessment and Accreditation of Laboratory Animal Care.

Preparations of immunogens (10E8-GT10.2 12mer (GT10.2, *n* = 14 mice) or negative-control 10E8-GT9-KO 12mer (KO, *n* = 8 mice)) at 5 µg per mouse were diluted in PBS at a volume of 100 µl per mouse for intraperitoneal injection and mixed at a 1:1 ratio with 2% alhydrogel (Invitrogen) for at least 20 min. The final formulation was injected (total volume of 200 µl per mouse).

Six weeks after immunization, mice were killed, and whole spleens were mechanically dissociated to generate single-cell suspensions. ACK lysis buffer was used to remove red blood cells, and splenocytes were then resuspended in FACS buffer (2% FBS/PBS), Fc blocked (clone 2.4G2, BD Biosciences) and stained for viability with Live/Dead Blue (Thermo Fisher Scientific) for 20 min at 4 °C. For surface staining, 10E8-GT10 probes (described above) and the following antibodies were used: CD4-APC-eF780, CD8-APC-eF780, Gr-1-APC-eF780, F4/80-APC-eF780, B220-BUV395, CD95-PE-Cy7, CD38-A700, CD45.1-PerCP-Cy5.5, CD45.2-PE and IgD-BV421. Cells were acquired on a BD LSRFortessa (BD Biosciences) for flow cytometric analysis and sorted using a BD FACS Aria II instrument (BD Biosciences). Data were analyzed using FlowJo software (TreeStar). B cells were single-cell dry sorted into 96-well PCR plates, rapidly frozen on dry ice and stored at −80 °C until processing.

Following single-cell sorting of antigen-specific B cells, the genes encoding the variable region of the heavy and light chains of IgG were amplified through reverse transcription PCR. In brief, first-strand cDNA synthesis was performed using SuperScript III Reverse Transcriptase (Invitrogen) according to manufacturer’s instructions. Nested PCR reactions consisting of PCR-1 and PCR-2 were performed as 25-µl reactions using HotStarTaq enzyme (Qiagen), 10 mM dNTPs (Thermo Fisher Scientific) and cocktails of *Igg*- and *Igk*-specific primers and thermocycling conditions described previously^73^. PCR products were analyzed on 2% 96-sample precast E-Gels with SYBR Safe (Thermo Fisher Scientific), and wells with bands of the correct size were submitted to GENEWIZ for Sanger sequencing. Heavy chain products were sequenced using the heavy chain reverse primer from PCR-2 (5′-GCTCAGGGAARTAGCCCTTGAC-3′), and the light chain was sequenced using the light chain reverse primer (5′-TGGGAAGATGGATACAGTT-3′) from PCR-2. Reads were quality checked, trimmed, aligned and analyzed using Geneious software (Biomatters). IMGT/V-QUEST (http://www.imgt.org)^74,75^ was used for mouse/human immunoglobulin gene assignments.

### hD3-3/J_H_6 rearranging mouse construction, characterization and immunization studies

To generate the hD3-3/J_H_6 mice, a cassette containing hD3-3 and hJ_H_6 (Extended Data Fig. 7) was integrated via homologous recombination into the mouse DQ52/J_H_ locus of an F1 (129/Sv × C57BL/6) embryonic stem (ES) cell line^76^. As a result, hD3-3 substitutes for mouse DQ52, and hJ_H_6 substitutes for mouse J_H_1-4. Correct integration of the hD3-3/J_H_6 cassettes was verified by Southern blotting. An ES clone with hD3-3/J_H_6 was injected into Rag2-deficient blastocysts to yield chimeric mice^77^, which were subsequently crossed with 129SVE mice to give germline transmission. For the B cell analysis shown in Extended Data Fig. 7b–e, splenocytes from wild-type mice were stained with the following antibodies: FITC anti-B220 and APC anti-Thy1.2 (Extended Data Fig. 7b); FITC anti-B220, PE anti-IgM and APC anti-IgD (Extended Data Fig. 7c); FITC anti-B220, APC anti-CD93, PE/Cy7 anti-CD23 and PerCP/Cy5.5 anti-CD21 (Extended Data Fig. 7d); and FITC anti-B220, biotin anti-IgK, APC streptavidin and PE anti-IgL (Extended Data Fig. 7e). Dead cells were gated out by Sytox blue staining. Flow cytometry was performed in a AttuneNxT instrument, and data were analyzed with FlowJo10 software. For IgH repertoire analysis shown in Extended Data Fig. 7f,g, genomic DNA was isolated from splenocytes of hD3-3/J_H_6 mice. Repertoire analysis was performed with the HTGTS-rep-seq technique^78,79^. Library synthesis was initiated with a primer downstream of hJ_H_6: 5Biosg/CAA CCT GCA ATG CTC AGG AA. Illumina MiSeq adaptors were added to the ends of library DNA, and sequencing was performed on a MiSeq instrument. Sequencing data were analyzed with the HTGTS-rep-seq pipeline.

Homozygous hD3-3/J_H_6 mice (>6 weeks old) were randomly distributed into groups. Mice were injected with 10 µg (50 µl total volume) of Moderna mRNA-LNPs^80,81^ encoding 10E8-GT12 24mer intramuscularly under anesthesia (5% isoflurane induction) in the left quadriceps muscle. All primes and subsequent boosts were performed in the same location. Protein injections (20 µg, 50 µl total volume) were performed subcutaneously (tail base) using 5 µg of SMNP^43^. Insulin syringes (BD) were used for all injections.

Mice were killed with compressed CO_2_ (100%) in a clear chamber to allow for visualization of respiration and subsequent death via respiratory cessation. Blood was collected from the chest cavity before removal of the spleen and lymph nodes (mesenteric, inguinal and popliteal; RNA injections only, left leg only). Tissues were placed in 3 ml of FACS buffer (1× PBS (calcium/magnesium free), 1 mM EDTA, 25 mM HEPES (pH 7.0) and 1% heat-inactivated FBS) in a 15-ml polypropylene tube on ice. Tissues were disassociated using the rough ends of two sandblasted microscope slides in a 5-ml Petri dish and returned to the same 15-ml polypropylene tube for centrifugation (460*g* for 5 min at 4 °C). Red blood cell lysis was performed using 1 ml of ACK buffer (Quality Biological) for 2 min on ice in a 15-ml polypropylene tube. Lysis was halted by adding 14 ml of FACS buffer per sample. After lysis and centrifugation (460*g* for 5 min), cells were resuspended in 3 ml of Bambanker freezing medium (Bulldog Bio) before filtration through a cotton-plugged, borosilicate Pasteur pipette into a borosilicate glass test tube. One milliliter of filtered cell solution was subsequently divided into three cryovials per mouse, which were precooled in a styrofoam rack on dry ice. Cells were stored at −80 °C for 2–7 days before long-term storage in liquid nitrogen. Sera were collected by spinning the blood at 14,000 rpm for 30 min. Sera were stored at −20 °C. All work followed IACUC guidelines associated with animal protocol number 20-0001.

For immunized samples, frozen splenocytes and lymphocytes were thawed in 10 ml of 50:50 heat-inactivated FBS (Omega Scientific, FB-02)/RPMI (Gibco, 61870-036) prewarmed to 37 °C. Unimmunized splenocytes for naive B cell sorting were used fresh after processing. Cells were spun down at 400*g* for 5 min. After the supernatant was removed, cells were resuspended in 3 ml of FACS buffer (1% (vol/vol) heat-inactivated FBS, 1 mM EDTA (Invitrogen, 15575-038) and 1 mM HEPES (Gibco, 15630-080) in DPBS (Corning, 21-031-CV)) and enumerated. After counting, cells were subjected to B cell isolation using a Stemcell EasySep Mouse Pan-B Cell Isolation kit (Stemcell, 19844A) according to manufacturer’s provided instructions.

Streptavidin-conjugated baits were prepared by combining biotinylated monomeric baits or Env trimer baits with fluorescent streptavidin at room temperature for at least 1 h in the dark. Wild-type baits were complexed with streptavidin-AF647 (Invitrogen, S21374) and streptavidin-BV421 (BioLegend, 405225). KO baits were conjugated with TotalSeq-C hashtagged streptavidin-PE (BioLegend, 405261). Baits were conjugated with streptavidin at a 4:1 (bait/streptavidin) ratio and used at a final bait concentration of 200 nM for staining.

Isolated B cells were transferred over to 15-ml conical tubes, washed once with FACS buffer and stained with 100 µl of antibody cocktail mix consisting of FITC anti-CD19 (BioLegend, 152404), BV786 anti-IgM (BD, 743328), PerCP-Cy5.5 anti-IgD (BD, 564273), APC-Cy7 anti-F4/80 (BioLegend, 123118), APC-Cy7 anti-CD11c (BD, 561241), APC-Cy7 anti-Ly-6C (BD, 557661), APC-H7 anti-CD8a (BD, 560182) and APC-H7 anti-CD4 (BD, 560181). All antibodies were used at a 1:100 dilution. The fluorescent streptavidin KO baits were first added to cells with the antibody master mix and stained for 15 min at 4 °C in the dark, followed by the addition of wild-type baits for an extra 30 min. During the addition of antibody master mix, a unique TotalSeq-C anti-mouse hashtag antibody (BioLegend) was added to each sample at a concentration of 2.5 µl per up to 20 million cells. Following antibody staining, 1 ml of 1:300 live/dead stain (LIVE/DEAD Fixable Aqua, Invitrogen, L34966) was added to each sample and incubated for an additional 15 min at 4 °C. At the end of staining, cells were washed with 10 ml of FACS buffer and resuspended in 500 µl of FACS buffer.

All samples were sorted on a BD FACSMelody using FACSChorus software. Single-color compensations were performed using C57BL/6 splenocytes with matched antibodies. For channels used for bait detection, cells were stained with biotinylated anti-CD19 (BioLegend, 115503), followed by secondary staining with the appropriate fluorescent streptavidin. Samples were filtered through a 35-µm mesh-cap FACS tube (Falcon, 352235) before being loaded on the sorter. A maximum of 15,000 cells was sorted using purity mode into a PCR plate well containing 20 µl of 0.2-µm-filtered FBS. Event rates were typically maintained at ~1,000 events per s and no more than 1,500 events per s to ensure high sorting efficiencies.

Sorted samples were prepared for BCR sequencing by the 10x Genomics Single Cell Immune Profiling platform. After cell sorting, DPBS was added up to near the top of the sample collection well (~100 µl) and gently mixed to dilute the FBS catch buffer. The plate was sealed, and cells were spun down for 2 min at 2,000 rpm, after which the excess buffer was removed except for the ~38 µl required for the 10x Genomics GEM reaction. Samples were processed according to manufacturer’s user guide for Chromium Next GEM Single Cell 5′ Reagent kits v2 (Dual Index) with Feature Barcoding with two previously described main modifications^82^. The number of PCR cycles in the cDNA amplification step was determined by assuming that only 20% of the total number of cells sorted would be recovered. This modification was made based on the observation that, on average, the number of unique paired BCR sequences recovered from the 10x Genomics platform was typically ~20% of the total number of cells sorted. In the second modification, the number of PCR cycles for each of the V(D)J amplification steps was increased to ten cycles if the number of cells sorted (according to the sorter) was fewer than 1,000 cells. Pooled libraries were sequenced on an Illumina NextSeq 2000 using NextSeq 1000/2000 Control Software and a 100-cycle P3 reagent kit (Illumina, 20040559) with a target depth of 5,000 paired-end reads for both the V(D)J and Feature Barcode Libraries and run using read parameters indicated in the 10x Genomics user guide.

Raw sequencing data were demultiplexed, processed into assembled V(D)J contigs and counts matrix files and assigned to specific animal IDs based on TotalSeq-C antibody hashtag counts using Cell Ranger (v6.1) and scab, as previously described^82^. Epitope-KO-positive cells were identified based on unique molecular identifier counts of the hashtagged PE-streptavidin probe using scab^82^. Gene assignment, annotation and formatting into Adaptive Immune Receptor Repertoire format^83^ for paired heavy and light chain antibody sequences were performed using SADIE with a custom hD3-3/J_H_6 mouse germline reference database^11^. SADIE outputs were summarized using custom Python scripts. Animals with low cell viability (<25,000 total live B cells) or low sequence recovery (<20 total sequences, where indicated <100 total sequences) were excluded. In addition, all non-IgG sequences were discarded. Sequences matching the criteria listed in Extended Data Fig. 1a were counted, and the frequencies were plotted in GraphPad Prism 9.5.1. Multipanel figures were assembled using Adobe Illustrator.

### Rhesus macaque repertoire characterization and immunization studies

Indian rhesus macaques (*Macaca mulatta*) for the 10E8-GT10.2 and MD39 immunization groups were housed at Alpha Genesis and maintained in accordance with National Institutes of Health (NIH) guidelines. This study was approved by the Alpha Genesis IACUC. All macaques were between 2 and 3 years old at the time of the immunization. For the 10E8-GT10.2 immunization group, eight macaques were used in the study, with four females and four males. Immunizations were given subcutaneously in the left and right deltoids with a total dose of 50 µg of 10E8-GT10.2 12mer nanoparticle and 375 µg of SMNP^43^ per side. For the MD39 control group, four macaques were used in the study, with two females and two males. Macaques were immunized subcutaneously in the left and right midthighs with a total dose of 50 µg of MD39 and 375 µg of SMNP^43^ per side. For priming, a seven-dose 12-day escalating dose strategy was used^42^. Data from the MD39 group have been previously published^50^.

For the 10E8-GT12 immunization group, macaques were housed at the Yerkes National Primate Research Center and were maintained in accordance with NIH guidelines. This study was approved by the Emory University IACUC. Six male macaques were used in the study and were aged between 3 and 4 years at the time of immunization. Immunizations were given subcutaneously in the left and right midthighs with a total dose of 50 µg of 10E8-GT12 12mer and 187.5 µg of SMNP-QS21 per side using a seven-dose 12-day escalating dose strategy^50^.

For the naive B cell screening study, macaques were housed at the Yerkes National Primate Research Center and were maintained in accordance with NIH guidelines. The study was approved by the Emory University IACUC. Blood was drawn from nine unimmunized macaques, processed for PBMCs and frozen and stored in liquid nitrogen until further analysis.

### Rhesus macaque lymph node fine needle aspiration

Lymph node fine needle aspirates were used to sample either the left or right draining axillary lymph nodes or the left and right draining inguinal lymph nodes, depending on the route of immunization. Draining lymph nodes were identified by palpation, and fine needle aspirates were performed by a veterinarian. A 22-gauge needle attached to a 3-ml syringe was passed into the lymph node up to five times. Samples were dispensed into RPMI medium containing 10% FBS and 1× pen/strep. ACK lysing buffer was used if the sample was contaminated with red blood cells. Lymph node fine needle aspirate samples were frozen and stored in liquid nitrogen until analysis.

### Rhesus macaque *IGHD3-3* genotyping

To genotype *IGHD3-3* (‘*IGHD3.41*’), we used targeted long-read Pacific Biosciences single-molecule real-time sequencing data generated for each macaque in the study cohort. Sequencing data were generated by adapting the published human immunoglobulin loci-targeted enrichment protocol^84,85^. Briefly, a custom oligonucleotide probe panel was designed (‘HyperExplore’, Roche) using immunoglobulin heavy chain (*IGH*), κ (*IGK*) and λ (*IGL*) genomic region sequences from the rhesus macaque genome reference build (RheMac10) and alternative haplotype assemblies from Cirelli et al.^42^ as sequence targets.

High-molecular-weight genomic DNA was isolated from PBMCs collected from each macaque using a DNeasy kit (Qiagen). DNA (1–2 µg) was then sheared using g-tubes (Covaris) and size selected using a Blue Pippin instrument (Sage Science). Size-selected DNA was end repaired and A-tailed using the standard KAPA library protocol (Roche), followed by ligation of sample-specific sequence barcodes and universal primers. PCR amplification was performed for eight to nine cycles using PrimeSTAR GXL polymerase (Takara), and the resulting products were further size selected and purified using 0.7× AMPure PB beads (Pacific Biosciences). Target enrichment hybridization was performed using *IGH*/*IGK*/*IGL*-specific oligonucleotide probes (Roche). Target fragments were recovered using streptavidin beads (Life Technologies), followed by a second round of PCR amplification for 16–18 cycles using PrimeSTAR GXL (Takara). Long-read sequencing libraries were prepared using a SMRTbell Express Template Preparation kit 2.0 (Pacific Biosciences), including Damage Repair and End Repair mix to repair nicked DNA, followed by the addition of an A-tail and overhang ligation with SMRTbell adapters. Libraries were then treated with a nuclease cocktail to remove unligated input material and purified with 0.45× AMPure PB beads (Pacific Biosciences). The resulting libraries were pooled and sequenced on the Sequel IIe system (2.0 chemistry; 30-h movies) to generate high-fidelity (‘HiFi’) reads, with average read accuracy 99.891325.

HiFi reads for each macaque were mapped to the RheMac10 genome reference. To genotype *IGHD3-3*, phased single-nucleotide variants representing two distinct alleles were resolved from HiFi reads spanning the *IGHD3-3* gene. At least ten representative HiFi reads were required to include a given allele in the genotype of an animal.

### Rhesus macaque flow cytometry and sorting

Frozen macaque fine needle aspiration or PBMC samples were thawed and recovered in R10. The cells were counted and stained with the appropriate staining panel. Fluorescently labeled antigen tetramer probes were prepared by incrementally mixing fluorophore-conjugated streptavidin with small volumes of biotinylated antigen probes in 1× PBS at room temperature over the course of 45 min. The KO probe, 10E8-GT10.2-KO or 10E8-GT12-KO, was first added to the cells for 20 min, followed by the addition of either 10E8-GT10.2 or 10E8-GT12 for 30 min and then with the surface antibodies for 30 min at 4 °C, similar to previously described protocols^42,50^. For samples being sorted, anti-human TotalSeq-C hashtag antibodies (BioLegend) were added to each individual sample at a concentration of 2 µg per 5 million cells along with the surface antibody master mix. FBS (10%) in RPMI (R10) supplemented with 1× pen/strep and 1× GlutaMAX was used as the FACS buffer. All samples were either acquired or sorted on a FACSymphony S6 (BD Biosciences) using FACSDiva software. For sorted lymph node fine needle aspiration and PBMC samples, indexed V(D)J and Feature Barcode libraries were prepared according to the protocol for Single Index Chromium Next GEM Single Cell V(D)J Reagent kits v1.1 with Feature Barcode technology (10x Genomics). For other PBMC samples, indexed V(D)J, Feature Barcode and Gene Expression (GEX) libraries were prepared using the protocol for Dual Index Chromium Next GEM Single Cell 5′ Reagent kits v2 with Feature Barcode technology (10x Genomics). Custom primers designed to target rhesus macaque BCR constant regions were used at previously described concentrations^50^.

The following reagents were used for staining: AF647 streptavidin (Invitrogen), BV421 streptavidin (BioLegend), BV650 streptavidin (BioLegend), PhycoLink streptavidin-RPE (ProZyme), PE-Cy7 streptavidin (BioLegend), BV711 streptavidin (BioLegend), BUV737 streptavidin (BD Biosciences), eBioscience Fixable Viability Dye eFluor 506 (Invitrogen), mouse anti-human CD3 BV786, APC-Cy7 (SP34-2, BD Biosciences), mouse anti-human CD4 BV711 (OKT4, BioLegend), mouse anti-human CD8a APC-eFluor 780 (RPA-T8, Thermo Fisher Scientific), mouse anti-human CD14 APC-Cy7 (M5E2, BioLegend), mouse anti-human CD16 APC-eFluor 780 (eBioCB16, Thermo Fisher Scientific), mouse anti-human CD20 AF488, PerCP-Cy5.5 (2H7, BioLegend), mouse anti-human CD27 PE-Cy7, BV650 (O323, BioLegend), mouse anti-human CD38 APC (OKT10, NHP Reagents), mouse anti-human CD71 PE-CF594 (L01.1, BD Biosciences), mouse anti-human PD-1 BV605 (EH12.2H7, BioLegend), mouse anti-human CXCR5 PE-Cy7 (MU5UBEE, Thermo Fisher Scientific), goat anti-human IgD FITC, AF488 (polyclonal, Southern Biotech), mouse anti-human IgG AF700, BV786 (G18-145, BD Biosciences), mouse anti-human IgM PerCP-Cy5.5, BV605, BUV395 (G20-127, BD Biosciences) and TotalSeq-C anti-human Hashtag antibody 1-10 (LNH-94 and 2M2, BioLegend).

### Rhesus macaque BCR sequencing and processing

Cell Ranger v3.0.2 was used for assembly of full-length V(D)J reads. A custom rhesus macaque germline V(D)J reference was generated using previously published databases^41,42,86^. The constants.py file within the Cell Ranger Python library was modified to increase the CDR3 maximum length to 110 nucleotides. For samples where a GEX library was generated, Cell Ranger v3.1 was used to obtain the gene expression matrix from these sequenced GEX libraries. The libraries were aligned to the Ensemble Mmul10 reference genome, with the addition of mitochondrial genes from Mmul9. Sequences were demultiplexed by the hashtag read counts using the MULTIseqDemux command in Seurat v4 (ref. ^87^). Sequences were further processed using the same pipeline described above for experiments in DJ mice, except using the default macaque germline reference database and searching for macaque homologs of 10E8-class and LN01-class V_H_ genes as listed in Supplementary Table 5.

### Neutralization assays

Neutralization assays were performed using the TZM-bl assay, as described previously^88,89^.

### Statistical analyses

Statistical analysis using the indicated tests and plotting of all data were performed using GraphPad Prism 9.5.1. No statistical methods were used to predetermine sample sizes, but our sample sizes are similar to those reported in previous publications^7,33,45,47,51^. Data collection and analysis were not performed blind to the conditions of the experiments

### Reporting summary

Further information on research design is available in the Nature Portfolio Reporting Summary linked to this article.

## Online content

Any methods, additional references, Nature Portfolio reporting summaries, source data, extended data, supplementary information, acknowledgements, peer review information; details of author contributions and competing interests; and statements of data and code availability are available at 10.1038/s41590-024-01833-w.

### Supplementary information

Reporting Summary

Supplementary Table 1 Sequences of antibodies synthesized in this study.

Supplementary Table 2 Monovalent affinities of *K*_d_ values determined by SPR.

Supplementary Table 3 Sequences of human BCRs sorted with 10E8-GT scaffolds.

Supplementary Table 4 Sequences of BCRs induced by 10E8-GT nanoparticles in hD3-3/J_H_6 mice.

Supplementary Table 5 Sequences of BCRs induced by 10E8-GT nanoparticles in rhesus macaques.

Supplementary Table 6 Neutralization of mAbs induced by 10E8-GT nanoparticles in hD3-3/J_H_6 mice and rhesus macaques.

## Data Availability

BCR sequences from human naive B cells and immunized mice and macaques are available in Supplementary Tables 3–5 and the public data repository https://github.com/SchiefLab/10E8 permanently archived at 10.5281/zenodo.11003090 (ref. ^90^). Atomic coordinates and structure factors have been deposited in the PDB with the following accession codes: 8TZW (10E8-GT4 + 10E8-iGL1), 8U03 (10E8-GT10.1 + 10E8-NGS-03), 8U08 (10E8-GT11 + 10E8-iGL1), 8TZN (10E8-GT10.2 + NHP W3-01), 8V2E (10E8-B1 + 10E8) and 8SX3 (10E8-GT10.2 + 10E8 + DJ-W6-10). Three-dimensional EM reconstructions are available from the Electron Microscopy Data Bank under accession code EMD-40825.
