# Author Correction: Vaccination induces broadly neutralizing antibody precursors to HIV gp41

**DOI:** 10.1038/s41590-024-01891-0

**Published:** 2024-06-14

**Authors:** Torben Schiffner, Ivy Phung, Rashmi Ray, Adriana Irimia, Ming Tian, Olivia Swanson, Jeong Hyun Lee, Chang-Chun D. Lee, Ester Marina-Zárate, So Yeon Cho, Jiachen Huang, Gabriel Ozorowski, Patrick D. Skog, Andreia M. Serra, Kimmo Rantalainen, Joel D. Allen, Sabyasachi Baboo, Oscar L. Rodriguez, Sunny Himansu, Jianfu Zhou, Jonathan Hurtado, Claudia T. Flynn, Katherine McKenney, Colin Havenar-Daughton, Swati Saha, Kaitlyn Shields, Steven Schultze, Melissa L. Smith, Chi-Hui Liang, Laura Toy, Simone Pecetta, Ying-Cing Lin, Jordan R. Willis, Fabian Sesterhenn, Daniel W. Kulp, Xiaozhen Hu, Christopher A. Cottrell, Xiaoya Zhou, Jennifer Ruiz, Xuesong Wang, Usha Nair, Kathrin H. Kirsch, Hwei-Ling Cheng, Jillian Davis, Oleksandr Kalyuzhniy, Alessia Liguori, Jolene K. Diedrich, Julia T. Ngo, Vanessa Lewis, Nicole Phelps, Ryan D. Tingle, Skye Spencer, Erik Georgeson, Yumiko Adachi, Michael Kubitz, Saman Eskandarzadeh, Marc A. Elsliger, Rama R. Amara, Elise Landais, Bryan Briney, Dennis R. Burton, Diane G. Carnathan, Guido Silvestri, Corey T. Watson, John R. Yates, James C. Paulson, Max Crispin, Gevorg Grigoryan, Andrew B. Ward, Devin Sok, Frederick W. Alt, Ian A. Wilson, Facundo D. Batista, Shane Crotty, William R. Schief

**Affiliations:** 1 Department of Immunology and Microbiology, The Scripps Research Institute, La Jolla, CA USA; 2 IAVI Neutralizing Antibody Center, The Scripps Research Institute, La Jolla, CA USA; 3 Center for HIV/AIDS Vaccine Immunology and Immunogen Discovery (CHAVD), The Scripps Research Institute, La Jolla, CA USA; 4 Institute for Drug Discovery, Leipzig University Medical Faculty, Leipzig, Germany; 5 Division of Vaccine Discovery, La Jolla Institute for Allergy and Immunology, La Jolla, CA USA; 6 The Ragon Institute of Mass General, MIT and Harvard, Cambridge, MA USA; 7 Department of Integrative Structural and Computational Biology, The Scripps Research Institute, La Jolla, CA USA; 8 Howard Hughes Medical Institute, Program in Cellular and Molecular Medicine, Boston Children’s Hospital, Boston, MA USA; 9 Department of Genetics, Harvard Medical School, Boston, MA USA; 10 School of Biological Sciences, University of Southampton, Southampton, UK; 11 Department of Molecular Medicine, The Scripps Research Institute, La Jolla, CA USA; 12 Department of Biochemistry and Molecular Genetics, University of Louisville School of Medicine, Louisville, KY USA; 13 Moderna, Inc., Cambridge, MA USA; 14 Department of Computer Science, Dartmouth College, Hanover, NH USA; 15 Division of Microbiology and Immunology, Emory National Primate Research Center, Emory University, Atlanta, GA USA; 16 Department of Microbiology and Immunology, Emory School of Medicine, Atlanta, GA USA; 17 Multi-omics Vaccine Evaluation Consortium, The Scripps Research Institute, La Jolla, CA USA; 18 San Diego Center for AIDS Research, The Scripps Research Institute, La Jolla, CA USA; 19 Department of Pathology and Laboratory Medicine, Emory University School of Medicine, Atlanta, GA USA; 20 Department of Biological Sciences, Dartmouth College, Hanover, NH USA; 21 Department of Biology, Massachusetts Institute of Technology, Cambridge, MA USA; 22 Division of Infectious Diseases, Department of Medicine, University of California San Diego, La Jolla, CA USA; 23 Present Address: Generate Biomedicines, Inc., Somerville, MA USA

**Keywords:** Vaccines, X-ray crystallography, HIV infections

Correction to: *Nature Immunology* 10.1038/s41590-024-01833-w, published online 30 May 2024

In the version of the article initially published, Steven Schultze’s surname appeared incorrectly (as Schulze) and has now been amended in the HTML and PDF versions of the article.

