# Supplementary material for: Vaccination induces broadly neutralizing antibody precursors to HIV gp41
Source: Nat Immunol. 2024 May 30;25(6):1073–82. doi: 10.1038/s41590-024-01833-w (PMC11147780; doi:10.1038/s41590-024-01833-w)
Supplement: Supplementary file 1 — Reporting Summary [file 41590_2024_1833_MOESM1_ESM.pdf]

nature portfolio

Corresponding author(s): William R. Schief

Last updated by author(s): April 1st, 2024

Reporting Summary

Nature Portfolio wishes to improve the reproducibility of the work that we publish. This form provides structure for consistency and transparency in reporting. For further information on Nature Portfolio policies, see our Editorial Policies and the Editorial Policy Checklist.

Statistics

For all statistical analyses, confirm that the following items are present in the figure legend, table legend, main text, or Methods section.

n/a	Confirmed	
☐	☒	The exact sample size (*n*) for each experimental group/condition, given as a discrete number and unit of measurement
☐	☒	A statement on whether measurements were taken from distinct samples or whether the same sample was measured repeatedly
☐	☒	The statistical test(s) used AND whether they are one- or two-sided<br>*Only common tests should be described solely by name; describe more complex techniques in the Methods section.*
☒	☐	A description of all covariates tested
☐	☒	A description of any assumptions or corrections, such as tests of normality and adjustment for multiple comparisons
☐	☒	A full description of the statistical parameters including central tendency (e.g. means) or other basic estimates (e.g. regression coefficient) AND variation (e.g. standard deviation) or associated estimates of uncertainty (e.g. confidence intervals)
☐	☒	For null hypothesis testing, the test statistic (e.g. *F*, *t*, *r*) with confidence intervals, effect sizes, degrees of freedom and *P* value noted<br>*Give P values as exact values whenever suitable.*
☒	☐	For Bayesian analysis, information on the choice of priors and Markov chain Monte Carlo settings
☒	☐	For hierarchical and complex designs, identification of the appropriate level for tests and full reporting of outcomes
☒	☐	Estimates of effect sizes (e.g. Cohen's *d*, Pearson's *r*), indicating how they were calculated

Our web collection on statistics for biologists contains articles on many of the points above.

Software and code

Policy information about availability of computer code

Data collection	FACSChorus and FACSDiva for flow cytometry; Carterra Navigator 1.7.4.2623 and Protein Manager 3.10 for SPR; ASTRA 7.1.1.3 for light scattering; NextSeq 1000/2000 Control Software for NGS sequencing.
Data analysis	HKL2000, UCSF ChimeraX, Coot 0.9.8, Phenix and Rosetta were used for structure determination. Sequencher 5.0, Cell Ranger v3.0.2, CellRanger v6.1.2, Immcantation, IgBlast, SADIE, Geneious, IMGT/V-QUEST, scab and Seurat v4 were used to process sequencing data. Statistical analysis and plotting were performed in Graphpad Prism 9.5.1. Flow data were analyzed in FlowJo 10. Figures were assembled in Adobe Illustrator 27.5. Carterra kinetics 1.9.0.4156, Protein Manager 3.10 and R Studio were used to analyze SPR data. ASTRA 7.1.1.3 was used to analyze light scattering data.

For manuscripts utilizing custom algorithms or software that are central to the research but not yet described in published literature, software must be made available to editors and reviewers. We strongly encourage code deposition in a community repository (e.g. GitHub). See the Nature Portfolio guidelines for submitting code & software for further information.

Data

Policy information about availability of data

All manuscripts must include a data availability statement. This statement should provide the following information, where applicable:

- Accession codes, unique identifiers, or web links for publicly available datasets
- A description of any restrictions on data availability
- For clinical datasets or third party data, please ensure that the statement adheres to our policy

X-ray and Cryo-EM coordinates and structure factors are deposited in the RCSB Protein Data Bank under PDB ID: 8TZW, 8U03, 8U08, 8TZN, 8V2E (X-ray) and 8sx3 (Cryo-EM). Cryo-EM structures were deposited to the Electron Microscopy Data Bank under accession code EMD-40825. Previously published structures used for comparisons are available in the RCSB Protein Data Bank under PDB ID 4G6F and 5T85. BCR sequences from the OAS Observable Antibody Space database are accessible under https://opig.stats.ox.ac.uk/webapps/oas. BCR sequences from human naive B cells and immunized mice and NHPs are available in the public data repository https://github.com/SchiefLab/10E8 permanently archived at https://zenodo.org/badge/latestdoi/xxxxxxxxx.

Research involving human participants, their data, or biological material

Policy information about studies with human participants or human data. See also policy information about sex, gender (identity/presentation), and sexual orientation and race, ethnicity and racism.

Reporting on sex and gender	Not applicable
Reporting on race, ethnicity, or other socially relevant groupings	Not applicable
Population characteristics	Not applicable
Recruitment	Leukoreduction (LRS) tubes from healthy donor samples were purchased from the San Diego Blood Bank from consenting participants. Participants were not directly contacted or recruited, and therefore, there was no self-selection biases that may be present that will impact results.
Ethics oversight	Experiments including human samples were performed in accordance with protocols approved by the La Jolla Institute for Immunology (LJI) Institutional Review Board

Note that full information on the approval of the study protocol must also be provided in the manuscript.

Field-specific reporting

Please select the one below that is the best fit for your research. If you are not sure, read the appropriate sections before making your selection.

☒ Life sciences ☐ Behavioural & social sciences ☐ Ecological, evolutionary & environmental sciences

For a reference copy of the document with all sections, see nature.com/documents/nr-reporting-summary-flat.pdf

Life sciences study design

All studies must disclose on these points even when the disclosure is negative.

Sample size	Sample size in macaque immunizations was based on availability of Rhesus Macaques. For mouse experiments, the sample size of n=5 or n=6 per independent experiment was determined by animal availability and, for subsequent flow cytometry, availability of the cell sorter. This sample size is generally accepted in the field, as it allows for rigorous hypothesis testing, simultaneously keeping the number of animals as small as possible while still being able to meet the scientific objectives (as per the 3R and ARRIVE guidelines). Combined data from independent experiments are shown where available. SPR experiments were performed on all antibodies available at time of the experiment.
Data exclusions	Data obtained from frozen B cells were excluded if cell viability was low (<25000 total live B cells). In sequence analyses, animals with <20 total sequences or <100 sequence were excluded as indicated in the relevant figure legends.
Replication	Rhesus macaque immunizations were not repeated due to difficulty in having access to a large number of NHPs. Mouse immunizations were repeated and combined data from independent experiments are shown. Results from independent experiments were in good agreement. Human ex vivo sorting data were repeated and combined data from independent experiments are shown with good agreement between independent experiments. Some SPR experiments were repeated on a different instrument and results were in good agreement. Other experiments using LN FNA samples and PBMCs were performed once.
Randomization	Rhesus macaques from the first experiment were weight- and age-matched and randomly divided into groups. Mice were matched for age (>6 weeks and randomly divided into groups.
Blinding	Investigators were not blinded as no subjective measurement was involved

Reporting for specific materials, systems and methods

We require information from authors about some types of materials, experimental systems and methods used in many studies. Here, indicate whether each material, system or method listed is relevant to your study. If you are not sure if a list item applies to your research, read the appropriate section before selecting a response.

Materials & experimental systems

n/a	Involved in the study
☐	☒ Antibodies
☐	☒ Eukaryotic cell lines
☒	☐ Palaeontology and archaeology
☐	☒ Animals and other organisms
☒	☐ Clinical data
☒	☐ Dual use research of concern
☒	☐ Plants

Methods

n/a	Involved in the study
☒	☐ ChIP-seq
☐	☒ Flow cytometry
☒	☐ MRI-based neuroimaging

Antibodies

Antibodies used

10E8-class antibodies and other immunogen-specific antibodies described in this study were expressed in Freestyle 293F cells or ExpiCho cells and purified as described in the methods section. Sequences of these antibodies are listed in Supp. Table 1. Commericially available antibodies used in this study were: Mouse anti-human CD19 PE-Cy7 (HIB19, Thermo Fisher Scientific), mouse anti-human CD3 APC-eFluor 780 (UCHT1, Thermo Fisher Scientific), mouse anti-human CD14 APC-eFluor780 (61D3, Thermo Fisher Scientific), mouse anti-human CD16 APC-eFluor780 (eBioCB16, Thermo Fisher Scientific), mouse anti-human IgG APC-Cy7 (HP6017, BioLegend), mouse anti-human CD14 APC-Cy7 (M5E2, BioLegend), mouse anti-human IgG BV605 (G18-145, BD Biosciences), mouse anti-human IgD BUV395 (IA6-2, BD Biosciences), mouse anti-human CD27 BB515 (M-T271, BD Biosciences),CD4 Monoclonal Antibody (GK1.5) APC-eFluor 780 (Invitrogen, 47-0042-80), CD8a Monoclonal Antibody (53-6.7) APC-eFluor 780 (Invitrogen, 47-0081-80), F4/80 Monoclonal Antibody (BM8) APC-eFluor 780 (Invitrogen, 47-4801-80), Pacific Blue™ anti-mouse/human CD45R/B220 Antibody (Biolegend, 103227), PE-Cy7 Hamster Anti-Mouse CD95 (BD Biosciences, 557653), Anti-mouse CD38 Alexa700 (Invitrogen, 56-0381-82), PerCP/Cy5.5 anti-mouse CD45.1 (Biolegend, 110728), PE anti-mouse CD45.2 (Biolegend, 109808), FITC anti-CD19 (Biolegend, 152404), BV786 anti-IgM (BD, 743328), PerCP-Cy5.5 anti-IgD (BD, 564273), APC-Cy7 anti-F4/80 (Biolegend, cat # 123118), APC-Cy7 anti-CD11c (BD, 561241), APC-Cy7 anti-Ly-6C (BD, 557661), APC-H7 anti-CD8a (BD, 560182), APC-H7 anti-CD4 (BD, 560181), mouse anti-human CD3 BV786, APC-Cy7 (SP34-2, BD Biosciences), mouse anti-human CD4 BV711 (OKT4, BioLegend), mouse anti-human CD8a APC-eFluor780 (RPA-T8, Thermo Fisher Scientific), mouse anti-human CD14 APC-Cy7 (M5E2, BioLegend), mouse anti-human CD16 APC-eFluor780 (eBioCB16, Thermo Fisher Scientific), mouse anti-human CD20 Alexa Fluor 488, PerCP-Cy5.5 (2H7, BioLegend), mouse anti-human CD27 PE-Cy7, BV650 (O323, BioLegend), mouse anti-human CD38 APC (OKT10, NHP Reagents), mouse anti-human CD71 PE-CF594 (L01.1, BD Biosciences), mouse anti-human PD-1 BV605 (EH12.2H7, BioLegend), mouse anti-human CXCR5 PE-Cy7 (MU5UBEE, Thermo Fisher Scientific), goat anti-human IgD FITC, Alexa Fluor 488 (polyclonal, Southern Biotech), mouse anti-human IgG Alexa Fluor 700, BV786 (G18-145, BD Biosciences), mouse anti-human IgM PerCP-Cy5.5, BV605, BUV395 (G20-127, BD Biosciences).

The following reagents were used for staining in the human naïve B cell screening experiments: 1). 10E8-GT9.2: Alexa Fluor 647 Streptavidin (Invitrogen, S32357), BV421 streptavidin (BioLegend, 405225), PhycoLink Streptavidin-RPE (ProZyme, PJRS25), BB515 Streptavidin (BD Biosciences, 564453), mouse anti-human CD19 PE-Cy7 (HIB19, Thermo Fisher Scientific, 25-0199-42), mouse anti-human CD3 APC-eFluor 780 (UCHT1, Thermo Fisher Scientific, 47-0038-42), mouse anti-human CD14 APC-eFluor780 (61D3, Thermo Fisher Scientific, 47-0149-42), mouse anti-human CD16 APC-eFluor780 (eBioCB16, Thermo Fisher Scientific, 47-0168-42), mouse anti-human IgG APC-Cy7 (HP6017, BioLegend, 409314), and eBioscience Fixable Viability Dye eFluor 780 (Invitrogen, 65-0865-14). 2). 10E8-GT10.1 (single cell BCR amplification): Alexa Fluor 647 Streptavidin (Invitrogen, S32357), BV421 streptavidin (BioLegend, 405225), PhycoLink Streptavidin-RPE (ProZyme), PJRS25, mouse anti-human CD19 PE-Cy7 (HIB19, Thermo Fisher Scientific, 25-0199-42), mouse anti-human CD3 APC-eFluor 780 (UCHT1, Thermo Fisher Scientific, 47-0038-42), mouse anti-human CD14 APC-eFluor780 (61D3, Thermo Fisher Scientific, 47-0149-42), mouse anti-human CD16 APC-eFluor780 (eBioCB16, Thermo Fisher Scientific, 47-0168-42), mouse anti-human IgG APC-Cy7 (HP6017, BioLegend, 409314), and eBioscience Fixable Viability Dye eFluor 780 (Invitrogen, 65-0865-14). 3). 10E8-GT10.1 (10X Genomics): Alexa Fluor 647 Streptavidin (Invitrogen, S32357), BV421 streptavidin (BioLegend, 405225), PhycoLink Streptavidin-RPE (ProZyme, PJRS25), mouse anti-human CD19 PE-Cy7 (HIB19, Thermo Fisher Scientific, 25-0199-42), mouse anti-human CD3 APC-eFluor 780 (UCHT1, Thermo Fisher Scientific, 47-0038-42), mouse anti-human CD14 APC-eFluor780 (61D3, Thermo Fisher Scientific, 47-0149-42), mouse anti-human CD16 APC-eFluor780 (eBioCB16, Thermo Fisher Scientific, 47-0168-42), mouse anti-human IgG APC-Cy7 (HP6017, BioLegend, 409314), Propidium Iodide (PI, Thermo Fisher Scientific, 00-6990-42), and TotalSeq-C anti-human Hashtag antibody 5 (LNH-94 and 2M2, BioLegend). 4). 10E8-GT12: Alexa Fluor 647 Streptavidin(BioLegend, 405237), BV421 Streptavidin (BioLegend, 405225), PE Streptavidin (BioLegend, 405245), mouse anti-human CD3 APC-Cy7 (UCHT1, BioLegend, 300426), mouse anti-human CD14 APC-Cy7 (M5E2, BioLegend, 301820), mouse anti-human CD16 APC-Cy7 (3G8, BioLegend, 302018), mouse anti-human CD20 PE-Cy7 (2H7, BioLegend, 302312), mouse anti-human IgG BV605 (G18-145, BD Biosciences, 563246), mouse anti-human IgD BUV395 (IA6-2, BD Biosciences, 612798), mouse anti-human CD27 BB515 (M-T271, BD Biosciences, 564642), and eBioscience Fixable Viability Dye eFluor506 (Invitrogen, 65-0866-14).

The following reagents were used for staining in the NHP studies: Alexa Fluor 647 streptavidin (Invitrogen, S32357), BV421 streptavidin (BioLegend, 405225), BV650 streptavidin (BioLegend, 405232), PhycoLink streptavidin-RPE (ProZyme, PJRS25), PE-Cy7 streptavidin (BioLegend, 405206), BV711 streptavidin (BioLegend, 405241), BUV737 streptavidin (BD Biosciences, 612775), eBioscience Fixable Viability Dye eFluor 506 (Invitrogen, 65-0866-14), mouse anti-human CD3 BV786, APC-Cy7 (SP34-2, BD Biosciences, 563918, 557757), mouse anti-human CD4 BV711 (OKT4, BioLegend, 317440), mouse anti-human CD8a APC-eFluor780

	(RPA-T8, Thermo Fisher Scientific, 47-0088-42), mouse anti-human CD14 APC-Cy7 (M5E2, BioLegend, 301820), mouse anti-human CD16 APC-eFluor780 (eBioCB16, Thermo Fisher Scientific, 47-0168-42), mouse anti-human CD20 Alexa Fluor 488, PerCP-Cy5.5 (2H7, BioLegend, 302316, 302326), mouse anti-human CD27 PE-Cy7, BV650 (O323, BioLegend, 302838, 302828), mouse anti-human CD38 APC (OKT10, NHP Reagents, PR-3801), mouse anti-human CD71 PE-CF594 (L01.1, BD Biosciences, custom), mouse anti-human PD-1 BV605 (EH12.2H7, BioLegend, 329924), mouse anti-human CXCR5 PE-Cy7 (MU5UBEE, Thermo Fisher Scientific, 25-9185-42), goat anti-human IgD FITC, Alexa Fluor 488 (polyclonal, Southern Biotech, 2030-02, 2030-30), mouse anti-human IgG Alexa Fluor 700, BV786 (G18-145, BD Biosciences, 561296, 564230), mouse anti-human IgM PerCP-Cy5.5, BV605, BUV395 (G20-127, BD Biosciences, 561285, 562977, 563903), and TotalSeq-C anti-human Hashtag antibody 1-10 (LNH-94 and 2M2, BioLegend).
Validation	Characterization of antibodies produced in-house are shown in Supp. Table 2, Fig. 1, 2, 4 and Extended Data Fig. 4, 5, 8, 10. Antibodies used in FACS have been validated by their respective manufactures as detailed on their product information.

Eukaryotic cell lines

Policy information about cell lines and Sex and Gender in Research

Cell line source(s)	FreeStyle HEK293cells (ThermoFisher), ExpiCho cells (ThermoFisher)
Authentication	All cell lines were purchased from ThermoFisher and used without further authentication
Mycoplasma contamination	Cells were not tested for mycoplasma
Commonly misidentified lines (See ICLAC register)	No commonly misidentified cell lines were used in this study

Animals and other research organisms

Policy information about studies involving animals; ARRIVE guidelines recommended for reporting animal research, and Sex and Gender in Research

Laboratory animals	Indian rhesus macaques (Macaca mulatta), age 2-3 years were used for 10E8-GT10.2 immunization and control group. Indian rhesus macaques, aged 3-4 years were used for 10E8-GT12 immunization. F1 (129/Sv x C57BL/6) ES cells were used to create hD3-3/JH6 mice; C57BL/6J mice were used to create MPER18 mice; and B6.SJL-Ptprc a Pepc b /BoyJ mice were used for adoptive transfer experiments as detailed in the methods section. Mice were housed under specific pathogen free conditions at 68-72°F and 30-70% humidity, with 6pm-6am nocturnal dark light cycle.
Wild animals	No wild animals were used in the study.
Reporting on sex	10E8-GT10.2 immunizations were performed in 4 male and 4 female rhesus macaques; control animals included 2 male and 2 female rhesus macaques. 10E8-GT12 immunizations used all male animals due to limited animal availability. Male mice were used in adoptive transfer experiments, and both sexes of mice were used throughout the hD3-3/JH6 mouse studies. No significant differences were noted between sexes.
Field-collected samples	The study did not involve samples collected from the field.
Ethics oversight	All animal procedures and experiments were performed under the approval by the respective Institutional Animal Care and Use Committee (IACUC): Alpha Genesis Inc. IACUC (Rhesus macaque immunizations with 10E8-GT10.2 or control), Emory University IACUC (Rhesus macaque immunizations with 10E8-GT12), Scripps Research IACUC (hD3-3/JH6 immunization), Harvard University and the Massachusetts General Hospital IACUC (MPER18 mouse immunization).

Note that full information on the approval of the study protocol must also be provided in the manuscript.

Flow Cytometry

Plots

Confirm that:

☒ The axis labels state the marker and fluorochrome used (e.g. CD4-FITC).

☒ The axis scales are clearly visible. Include numbers along axes only for bottom left plot of group (a 'group' is an analysis of identical markers).

☒ All plots are contour plots with outliers or pseudocolor plots.

☒ A numerical value for number of cells or percentage (with statistics) is provided.

Methodology

Sample preparation	Cells from immunized hD3-3/J6 mice were disassociated using the rough ends of two sandblasted microscope slides followed by red blood cell lysis Ammonium Chloride-Potassium (ACK) buffer. Clarified cells were filtered through cotton-plugged, borosilicate Pasteur pipettes in Bambanker freezing medium and stored at -80°C for 2-7 days prior to long-term storage in liquid nitrogen. Whole spleens from mice in adoptive transfer experiments were mechanically dissociated to generate single-cell suspensions. ACK lysis buffer was used to remove red blood cells and splenocytes were then resuspended in FACS buffer

(2% FBS/PBS) for staining on the same day. Human PBMCs were isolated from blood by the LJI Blood Processing Core and were frozen down and stored in liquid nitrogen until analysis. Rhesus macaque LN FNAs were performed by a veterinarian. LNs were identified by palpation and a 22-gauge needle attached to a 3-mL syringe was passed into the LN up to 5 times. Samples were dispensed into RPMI media containing 10% fetal bovine serum (FBS) and 1x pen/strep. ACK buffer was used if the sample was contaminated with red blood cells. LN FNA samples were frozen and stored in liquid nitrogen until analysis. Staining of cells for flow cytometry was performed as detailed in the relevant methods sections.

Instrument	BD FACS Symphony S6 or BD FACSMelody cytometer
Software	BD FACSDiva, BD FACSChorus, FlowJo v.10 software
Cell population abundance	Information regarding cell abundance can be found in Fig. 3b-c, 4a-b, 4e and Extended data Fig. 6a-b, 6e-f, 8b, 10b-c.
Gating strategy	Cells were gated on the relevant lymphocyte population using FSC and SSC, doublets were excluded using scatter high vs areas, living cells were selected by negativity for the viability dye. Afterwards, relevant populations were gated depending on the analysis required. Complete gating strategies can be found in the Extended data (Extended data Fig. 4a, 8a, 9).

☒ Tick this box to confirm that a figure exemplifying the gating strategy is provided in the Supplementary Information.
